# Loss of the heterogeneous expression of flippase ATP11B leads to cerebral small vessel disease in a normotensive rat model

**DOI:** 10.1007/s00401-022-02441-4

**Published:** 2022-05-30

**Authors:** Sophie Quick, Tessa V. Procter, Jonathan Moss, Luise Seeker, Marc Walton, Angus Lawson, Serena Baker, Anna Beletski, Daniela Jaime Garcia, Mehreen Mohammad, William Mungall, Ami Onishi, Zuzanna Tobola, Michael Stringer, Maurits A. Jansen, Antoine Vallatos, Ylenia Giarratano, Miguel O. Bernabeu, Joanna M. Wardlaw, Anna Williams

**Affiliations:** 1 Centre for Regenerative Medicine, Institute for Regeneration and Repair, University of Edinburgh, Edinburgh, EH16 4UU UK; 2 Centre for Clinical Brain Sciences, Edinburgh Imaging, Row Fogo Centre for Research into Ageing and the Brain, University of Edinburgh, Edinburgh, EH16 4SB UK; 3 UK Dementia Research Institute, University of Edinburgh, Edinburgh, EH16 4SB UK; 4 College of Medicine and Veterinary Medicine, College of Science and Engineering, Bayes Centre, Usher Institute, University of Edinburgh, Edinburgh, EH16 4UX UK; 5 Bioresearch and Veterinary Services, University of Edinburgh, Edinburgh, EH16 4SB UK; 6 Centre for Cardiovascular Science, University of Edinburgh, Edinburgh, EH16 4SB UK

**Keywords:** Cerebral small vessel disease, Endothelial cell, Oligodendrocyte, ATP11B, Myelin, Heterogeneity

## Abstract

Cerebral small vessel disease (SVD) is the leading cause of vascular dementia, causes a quarter of strokes, and worsens stroke outcomes. The disease is characterised by patchy cerebral small vessel and white matter pathology, but the underlying mechanisms are poorly understood. This microvascular and tissue damage has been classically considered secondary to extrinsic factors, such as hypertension, but this fails to explain the patchy nature of the disease, the link to endothelial cell (EC) dysfunction even when hypertension is absent, and the increasing evidence of high heritability to SVD-related brain damage. We have previously shown the link between deletion of the phospholipase flippase *Atp11b* and EC dysfunction in an inbred hypertensive rat model with SVD-like pathology and a single nucleotide polymorphism (SNP) in *ATP11B* associated with human sporadic SVD. Here, we generated a novel normotensive transgenic rat model, where *Atp11b* is deleted, and show pathological, imaging and behavioural changes typical of those in human SVD, but that occur without hypertension. *Atp11b*KO rat brain and retinal small vessels show ECs with molecular and morphological changes of dysfunction, with myelin disruption in a patchy pattern around some but not all brain small vessels, similar to the human brain. We show that ATP11B/*ATP11B* is heterogeneously expressed in ECs in normal rat and human brain even in the same transverse section of the same blood vessel, suggesting variable effects of the loss of ATP11B on each vessel and an explanation for the patchy nature of the disease. This work highlights a link between inherent EC dysfunction and vulnerability to SVD white matter damage with a marked heterogeneity of ECs in vivo which modulates this response, occurring even in the absence of hypertension. These findings refocus our strategies for therapeutics away from antihypertensive (and vascular risk factor) control alone and towards ECs in the effort to provide alternative targets to prevent a major cause of stroke and dementia.

**Supplementary Information:**

The online version contains supplementary material available at 10.1007/s00401-022-02441-4.

## Introduction

Cerebral small vessel disease (SVD) is the leading cause of vascular dementia, is increasingly recognised as an important contributor to pathology in Alzheimer’s disease and causes 25% of ischemic stroke and most haemorrhagic strokes in older people [22, 53], making it a major target in tackling the global burden of neurodegenerative disease. SVD affects the small perforating blood vessels of the brain and is diagnosed using a collection of features on magnetic resonance imaging (MRI), including white matter hyperintensities (WMH), lacunes and microbleeds [56], with the increased extent of these white matter changes correlating with worse cognition [14, 19]. In addition to presenting with stroke or cognitive impairment, SVD may also cause impaired gait or balance, and neuropsychiatric symptoms including depression. However, the clinical expression is often ‘covert’ and under-recognised both by affected individuals and clinicians [26, 28, 41, 42]. Indeed, studies of the general population show that features of SVD are common, increase in prevalence with age and are associated with hypertension, diabetes and hypercholesterolaemia [50]. SVD is often considered to result primarily from hypertension [46], other vascular risk factors, or atherothromboembolic disease as in most large artery strokes. While hypertension is certainly a risk factor for developing SVD [54], approximately 30% of patients with sporadic SVD have no history of hypertension [29]. In addition, all common vascular risk factors combined only account for a small percentage of variance in WMH severity [53] and, consistent with this, it has been difficult to demonstrate that anti-hypertensive treatment can prevent worsening of MRI or clinical features [54].

This suggests that the risk of developing SVD is not simply a consequence of exposure to vascular risk factors in adulthood. Indeed, our epidemiological data show that increased SVD severity in later life associates with early life factors, such as lower cognitive ability in youth (and lower educational attainment) [4], independent of adult risk factor exposure [5]. Since cognitive ability is partly explained by white matter integrity [39], the association with SVD in later life may reflect inherently poorer white matter integrity from youth, and hence white matter vulnerability to damage [56]. Consistent with this, the integrity of normal appearing white matter is reduced in young adults who have an increased genetic risk for WMH, long before WMH develop [47].

The link between early life factors/white matter changes and vascular disease may lie with the endothelial cell (EC) of the blood–brain barrier. Using the inbred Spontaneously Hypertensive Rat–Stroke Prone strain (SHRSP), a well-established rat model with many features of sporadic human SVD [57–59], we previously demonstrated intrinsically dysfunctional ECs with reduced endothelial nitric oxide synthase (eNOS) and secondary white matter changes in young rats prior to onset of hypertension, due to block of oligodendroglial maturation through increased EC secretion of Heat Shock Protein 90 alpha (HSP90α) [44]. As these changes were also present in ex vivo SHRSP brain slices from postnatal rat pups after 3 weeks of culture (where there is no blood flow or pressure), this confirmed EC dysfunction was intrinsic, and that hypertension is not the sole cause of this SVD. Human post mortem brain tissue from the ‘Sudden Death’ brain bank, where pathology identified only early changes of SVD, also showed markers of endothelial dysfunction and oligodendroglial maturation block, confirming this pathological mechanism in humans [44]. By comparing the SHRSP whole genome sequence to that of its closest relative, the Spontaneous Hypertensive Rat (SHR) (which is hypertensive without SVD changes), we identified a homozygous exonic deletion mutation in *Atp11b* in the SHRSP but not in the SHR. This deletion caused global loss of *Atp11b* in all cells, but loss or knock down of *Atp11b/ATP11B* in cultured rodent or human ECs was sufficient to phenocopy the SHRSP EC dysfunction and secondary OPC maturation block [44]. *Atp11b* encodes a phospholipid flippase thought to be involved in membrane function and vesicular transport [38], not previously associated with human diseases, but we identified a SNP in *ATP11B* that associated with human WMH (a surrogate for SVD) in the CHARGE consortium [44].

This combined evidence led to our hypotheses that 1) ATP11B loss or modulation causes EC dysfunction and leads to changes reminiscent of SVD and 2) hypertension is not required for SVD pathology. Here, we test both hypotheses in a newly generated transgenic *Atp11b*KO rat model, a global knock-out (KO) designed to mirror the SHRSP but to be normotensive.

## Methods

### Generation of *Atp11b*KO rat

The rat *Atp11b* gene is located on rat chromosome 2 (GenBank accession number: XM_017591376.1; Ensembl: ENSRNOG00000052116) with the ATG start codon in exon 1 and TAG stop codon in exon 30. KO rat generation was designed by and purchased from Cyagen Biosciences Inc. The rat strain used was the standard Sprague–Dawley background rather than Wistar–Kyoto as in the SHRSP for ease of molecular genetics. Exons 5 to 8 were selected as target sites and guide RNAs chosen: gRNA1 (matches reverse strand of gene): ACAAAATGCTCACGCATCGAAGG gRNA2 (matches forward strand of gene): AGTGTAAAACTGTCATCGGCTGG. Cas9 and gRNAs were injected into fertilised eggs for KO production and screened for deletion of this segment using PCR primers: Rat *Atp11b*-pair1-F: GGGTGCTCTAACTGCTCCAAGGTT Rat *Atp11b*-pair1-R: TAGATGCTGAAACAGACAAAAGCTACCAC PCR product size Rat *Atp11b*-pair1, giving a wildtype allele fragment of 7050 bp and a mutant allele fragment of ~ 700 bp, thus deleting ~ 6350 bp. Two F0 founders were generated**,** RatID#18—male—missing 6352 bases on 1 allele and 6342 on other allele and Rat-ID#28—female—missing 6349 bases on one allele only. These were bred with WT rats, leading to 6 F1 rats, four missing 6352 bases on 1 allele (from Rat-ID#18) and 2 missing 6349 on 1 allele (from Rat-ID#28). These deletions lead to predicted truncated amino acid sequences which are identical MWRWVRQQLG FDPPHQSDTR TIYIANRFPQ NGLYTPQKFI DNRIISSKYT VWNFVPKNLF EQFRRVANFY FLIIFLVQLM IDTPTSPITS GLPLFFVITV TAIKQTSGTR ESLASWSQIK KYKRNFWCGC IHWDGNKDGI ELQEQVTEKI CCREINEHIF NNLSNNPYF CCREINEHIF NNLSNNPYF. Therefore, we considered these the same, and formed the KO rat by interbreeding the F1 generation. Off target analysis identified three potential off-target sites for gRNA1 and two for gRNA2, but sequenced PCR amplicons around these sites showed no mutations (data available on request).

### Housing and husbandry

All work was carried out under UK Home Office project licence PADF15B79. *Atp11b*KO animals were bred as heterozygous breeding pairs to generate WT and KO littermates for in vivo work. For neonatal cell isolation, WT/WT and KO/KO breeding pairs were used to ensure correct genotype at birth. We followed ARRIVE2 guidelines to minimize bias, with mixed-sex groups, and age-matching for WT and KO. Sample sizes were selected based on power calculations, animals were only excluded from samples when culls were essential—such as injury from fighting. Analysis was performed blinded to genotype and age. Animals were allowed to age to a maximum of 1 year, the maximum age permitted under our animal licence.

### Termination

For OPC isolation, pups at postnatal days 0–2 were terminated by an overdose of Euthatal (150 mg/kg; Merial) administered intraperitoneally. For brain microvascular ECs (BMECs), pups at postnatal days 5–7 were terminated by a rising concentration of carbon dioxide (CO_2_). For extraction of protein lysates, where fresh frozen tissue was required, animals were terminated by an overdose of Euthatal (150 mg/kg) and brains extracted and stored in PBS temporarily until processed. For immunohistochemistry (IHC), animals were first anaesthetized with Isoflurane by inhalation, then by intraperitoneal injection with Ketamine (Vetalar®) and Medetomidine (Domitor®). Intracardiac perfusion fixation was performed, first with phosphate buffered saline (PBS) then with 4% paraformaldehyde (PFA; Sigma-Aldrich; diluted in PBS (Gibco)).

### Blood pressure measurement

Blood pressure was measured weekly using The CODA™ Non-Invasive Blood Pressure System, and body weight was also recorded. Animals were habituated to the tube in advance to avoid stress. For each weekly reading, five test cycles were carried out before 20 recorded cycles for each animal. Readings were excluded by the system and repeated if they did not fit the expected curve. A weekly blood pressure average for each animal was obtained from a minimum of 8 recorded cycles. Experiments were carried out by a researcher blinded to genotype. Raw data was extracted from the operating systems by a different researcher and processed.

### Behavioural tests

Animals were handled prior to testing to minimize stress. The protocols were carried out by an independent researcher, who was blind to genotype to prevent experimenter bias.

### Open field and elevated maze

Open field or elevated maze was set up directly above the camera and AnyMaze software used to record when animals entered the centre of the field and the time spent in the edges. The animal was initially placed in the corner facing the wall for open field and placed in middle of the elevated maze. The open field test was run for 20 min, and the elevated maze test for 5 min, with the researcher hidden and blinded to genotype.

### Catwalk

Experiments were based on published methods with minor modifications [16, 17]. The automated gait analysis apparatus (Noldus, Wageningen, Netherland) consists of a glass plate walkway equipped with light-emitting diodes (LEDs). Light from the LEDs is emitted inside the glass plate, internally reflected, and refracted to the glass plate except in the area, where the animal's paw makes contact with the plate. A high-speed colour camera positioned underneath the glass plate captures images of the illuminated area and sends the imaging information to a computer that runs the analysis system (CatWalk XT version 10.6 software; Noldus). Animals walked on the glass plate walkway 3 times on the day before evaluation to habituate them. A satisfactory measured walk was defined as an uninterrupted walk across the walkway that left > 3 footprints for each hind limb. Recordings were repeated until three satisfactory walks were obtained.

### Novel object recognition

Experiments were based on published methods with minor modifications [30]. The test area consisted of an open field set up directly below the camera and AnyMaze software used to record when animals entered areas surrounding objects. After habituation to both the test area and familiarisation to two identical objects (two lego towers), a new object (lightbulb) replaced one of the original objects and the animal was initially placed in the corner facing the wall of the open field when recording was started. The percentage time exploration preference was calculated as a ratio: time spent exploring the novel object/time spent exploring the familiar object.

### Magnetic resonance imaging acquisition and processing

In vivo MRI data was acquired in a longitudinal way on age-matched *Atp11b*KO and Sprague–Dawley rats at both 3–4 months and at 9–10 months of age. Anaesthesia was induced in an adapted chamber with 4% isoflurane and a 30:70 O_2_/N_2_O ratio. Animals were then transferred to the MRI instrument cradle. A hot air blower was used to regulate physiological temperature (37 ± 1 °C). The head was secured laterally by conical ear rods and longitudinally by the nose cone used for anaesthetic gas delivery. The animals breathed spontaneously through a facemask, with isoflurane delivered at a constant flow mixed with a 40:60 ratio of O_2_/N_2_O (1 L min-1). Isoflurane concentration varied (1.5–3%) to maintain stable respiration rates (40–100 bpm). Respiration was monitored using a pressure sensor connected to an air-filled balloon placed under the animal abdomen. On completion of imaging, isoflurane delivery was stopped and animals observed until full recovery.

Images were acquired in a 400-mT/m gradient set 7 T Bruker scanner (Bruker Medical GmbH, Germany) with an 86-mm ID quadrature radiofrequency coil for transmission and a 4-channel phased array coil for reception. The acquisition parameters are listed in the table below (Table 1). A localizer scan was performed to ensure correct positioning before T_1_-weighted (T1W), T_2_-weighted (T2W), T_2_^*^-weighted (T2^*^W) and T2W with fluid attenuated inversion recovery (FLAIR) images were acquired from the caudal to cranial cerebral cortex. Images were uploaded to Carestream Vue PACS (Carestream Health, Onex, Canada) for quantitative and qualitative image assessment.

**Table 1:** FOV: field-of-view; SLT: slice thickness; TE: echo time; TR: repetition time; RARE: rapid acquisition with relaxation enhancement; FLASH: fast low angle shot; TI: inversion time

Protocol	Parameters	Resolution
T1W RARE	FOV 30 × 30 mm, Matrix 256 × 256 × 30, SLT 0.5 mm, TE 11.3 ms, TR 1200 ms	0.12 × 0.12 × 0.50 mm
T2W RARE	FOV 30 × 30 mm, Matrix 256 × 256 × 30, SLT 0.5 mm, TE 45 ms, TR 2500 ms	0.12 × 0.12 × 0.50 mm
T2*W FLASH	FOV 30 × 30 mm, Matrix 256 × 256 × 30, SLT 0.5 mm, TE 10 ms, TR 700 ms	0.12 × 0.12 × 0.50 mm
T2W FLAIR	FOV 30 × 30 mm, Matrix 192 × 192 × 15, SLT 1.0 mm, TE 45 ms, TR 8000 ms, TI 1860 ms	0.16 × 0.16 × 1.0 mm

Quantitative assessment was performed on T2W images using the graphics line measurement tool. Corpus callosum was measured in the coronal plane at three locations of one slice rostral to the merging of the anterior parts of the anterior commissure. Brain tissue volumes were measured using the 3D segmentation tool in ITK-SNAP [61]. Percentage tissue volume was calculated over 3 coronal slices by dividing tissue volume by total brain volume. Qualitative assessments for ventriculomegaly and potential microbleeds were performed using a combination of the four sequences acquired with particular emphasis on T2*W for microbleeds and FLAIR for ventriculomegaly. Methods were adapted from our human-based methods [51].

### RNA sequencing data

Single nuclei (sn) RNAsequencing data for normal adult human CNS ECs were subsetted from our data set as described [49] with the metadata described in Table 2.

**Table 2:** Donor information for human samples

individual ID	Sex	Age	PMI (h)	Cause of death	Neuropathological examination
SD026/16	F	37	126	Myocardial fibrosis	No significant abnormality
SD031/15	F	40	89	Hemopericardium	No significant abnormality
SD041/19	F	40	72	Unascertained	Mild cerebral amyloid angiopathy only. No significant acute pathology is identified
SD016/13	F	44	70	Unascertained	No significant abnormalities, in particular, no cause for sudden death
SD017/13	F	45	93	Coronary artery atherosclerosis	No significant abnormality and no pathology to account for sudden death
SD038/17	M	34	99	Ischaemic heart disease	No significant abnormality
SD022/16	M	39	86	Ischaemic heart disease	No significant abnormality
SD038/16	M	39	76	Suspension by ligature	No significant abnormality
SD029/17	M	40	103	Myocardial infarction	No significant abnormality
SD046/16	M	42	103	Suspension by ligature	Diffuse plaque deposition but no significant abnormalities
SD028/09	M	50	46	Ischaemic heart disease with coronary artery atherosclerosis	No significant abnormality
SD011/18	F	61	80	Multi-organ failure secondary to sepsis	No significant abnormality
SD008/17	F	71	96	Ischaemic and hypertensive heart disease	No significant abnormality
SD012/17	F	71	95	Plastic bag suffocation	Very focal Tau pathology (Braak stage 1) but no other significant abnormality
SD042/18	F	73	74	Hypertensive heart disease	No significant abnormality
SD014/13	F	74	41	Pulmonary thromboembolism	Mild degree of small vessel disease but no other significant abnormality
SD030/18	M	63	115	Ruptured atherosclerotic abdominal aortic aneurysm	No significant abnormality
SD042/14	M	63	76	Complications of metastatic renal cell carcinoma	Mild small vessel disease only. No metastatic/non-metastatic complications of renal cell carcinoma
SD036/17	M	71	71	Hemopericardium	No significant abnormality
SD024/17	M	72	60	Myocardial infarction	No significant abnormality
SD025/17	M	73	66	Ischaemic heart disease with cardiac enlargement	No significant abnormality

PMI: post-mortem interval in hours

We used scSorter [21] with weighted markers (Table 3) for blood vessel type annotation into venule, capillary and arteriole and examined *ATP11B* expression within each EC. Code available at link below.

**Table 3:** Markers for allocating ECs to vessel types using scSorter

Type	Marker	Weight
Capillary	MFSD2A	2
Capillary	SLC16A1	2
Capillary	SLC7A5	2
Capillary	TFRC	2
Capillary	ABCB1	2
Arterial	MGP	5
Arterial	VEGFC	5
Arterial	HEY2	5
Arterial	BMX	5
Arterial	EFNB2	5
Vein	ICAM1	5
Vein	IL1R1	5
Vein	EPHB4	5
Vein	NR2F2	2
Vein	VCAM1	5

### Preparation of brain ECs

As adapted from a previously published method [1], brains were extracted from rats aged postnatal days 5–7 and placed in working buffer (Hank’s balanced salt solution (HBSS) (Life Technologies) with 0.5% chromatographically purified bovine serum albumin (BSA) (First Link UK), 0.5% HEPES (Gibco), 0.5% pen/strep). Meninges and cerebellum were removed and brains were homogenised in 5 ml working buffer with a Dounce tissue grinder. Homogenate was isolated from the buffer by centrifugation for 5 min at 1800 revolutions per minute (RPM) at 4 °C. Blood vessel fragments were pelleted from this homogenate by centrifugation in 22% BSA for 15 min at 3000 RPM at 4 °C. The pellet of blood vessel fragments was kept on ice in working buffer, while the remainder was centrifuged again, for a total of 5 spins. Blood vessel fragments were pipetted onto an upside down 70 μm cell strainer to remove single cells then washed into a pooled mixture with working buffer. This was spun down (5 min at 1800 RMP, 4 °C) and the pellet then digested in 10 ml collagenase/dispase (1 mg/ml; Roche) and DNase I type IV (40 μg/ml; Sigma-Aldrich) for 45 min at 37 °C with regular agitation.

The resultant dissociated ECs were washed in working buffer and plated at 500,000 cells/well on collagen IV and fibronectin (100 μg/ml and 50 μg/ml, respectively; Sigma-Aldrich; overnight, 4 °C) coated coverslips in a 24-well plate in 1 ml Endothelial Growth Media-2 Bullet Kit (EGM-2; Lonza; Endothelial basal media-2 with 10% Fetal Bovine Serum (FBS), 0.4% hFGF-beta, 0.1% hEGF, 0.1% VEGF, 0.1% R3-IGF-1, 0.1% Ascorbic acid, 0.1% Gentamycin/Amphotericin-B, 0.04% hydrocortisone and 0.1% heparin) with 0.1% puromycin (Sigma-Aldrich; 5 mg/ml in Dulbecco’s phosphate buffered saline (DPBS; Sigma-Aldrich) first 2 days of culture only). Cells were grown in 7.5% carbon dioxide (CO_2_) and medium was changed every 2–3 days. Cultures were used in experiments once they had regions of confluence (before 10 days).

### Preparation of OPCs

As adapted from a previously published method [33], brains were extracted from rats aged postnatal days 0–2 and cortices isolated in MEM. Meninges were removed then cortices were minced with fine scissors and digested in MEM with papain (1.2U/ml; Worthington), l-cysteine (0.24 mg/ml; Sigma-Aldrich) and DNase I type IV (40 μg/ml) for 1 h at 37 °C. Cells were diluted in culture medium (Dulbecco’s modified Eagle medium (DMEM; Gibco) with 10% FBS (Gibco), 1% pen/strep), spun down (5 min at 1000 RPM), then resuspended with a 1 ml pipette. Cells were resuspended in culture medium (1.5 brains in 10 ml per flask) and grown in vented T75 flasks coated with poly-d-lysine (PDL; Sigma-Aldrich; 5 μg/ml in water; minimum 1 h coating, 37 °C) in 7.5% CO_2_.

10–14 days after dissection, the vented cap of the flasks was sealed and the flasks were placed on an orbital shaker at 240 RPM at 37 °C. After 1 h, the medium was removed (containing loosely attached microglia), fresh medium added, and the flask returned to the shaker for 16–18 h. This medium was removed and plated on 10 cm plastic petri dishes for 20–25 min (to remove remaining microglia) after which the cells were spun down (5 min at 1000 RPM). Cells were resuspended in 5 ml culture medium, gently triturated through a 21 gauge needle, counted, and plated at a density of 50,000 cells in a single droplet of SATO medium (DMEM with 1% pen/strep. 1% ITS supplement (Sigma-Aldrich), 16 μg/ml putrescine (Sigma-Aldrich), 400 ng/ml l-thyroxine (Sigma-Aldrich), 400 ng/ml Tri-iodothyroxine (Sigma-Aldrich), 60 ng/ml progesterone (Sigma-Aldrich), 100 μg/ml BSA (fraction V) (Sigma-Aldrich)) per PDL coated coverslip in a 24 well plate, topped up to 0.5 ml SATO medium per well after 20 min at 37 °C for cells to stick down. OPCs were grown for 2 days and the medium was not changed during this time.

### Conditioned media experiments

ECs were grown in their normal culture medium until reaching confluence and forming tight junctions (< 10 day in vitro). At this point the media was changed and this was conditioned for 2–3 days. Media was then removed and stored at − 20 °C until required. When defrosted, conditioned media was mixed at a 1:1 ratio with SATO media. Cells grown in conditioned media were prepared as normal. Conditioned media was added to OPCs after the step at which they were left for 20 min to stick down. Cells were left for 3 days in conditioned media before being fixed and analysed.

### Immunofluorescence

Brains were extracted from animals after perfusion fixation and incubated for no more than 4 h in 4% PFA at 4 °C. PFA-fixed brains were then incubated first in 15% sucrose (overnight, 4 °C; Sigma-Aldrich) and then in 30% sucrose (overnight, 4 °C). Sections were cut at 10 μm thickness using a cryostat and collected in series so that each slide for immunostaining had a spread of coronal sections of the deep white matter.

Eyes were extracted following perfusion and fixation and washed with PBS before storage in 30% w/v sucrose. Eyes were dissected using an adapted version of a previously published method [13]. Immunofluorescence staining was performed with the retina still attached to the sclera of the back of the eye due to tissue fragility. Following staining, the retina was quartered and the sclera discarded before flat-mounting for imaging. These were imaged with a Zeiss wide-field microscope.

For rat brain and cell immunofluorescence: where necessary for antibodies, antigen retrieval was carried out by boiling sections for 10 min in a microwave in 0.01 M citrate buffer (Vector Laboratories). Sections were then cooled for 20 min in running tap water. Sections were blocked for at least 1.5 h at room temperature in blocking solution (10%v/v heat inactivated horse serum (HIHS), 0.5%v/v triton (Fisher) in PBS). Primary antibodies, diluted in blocking solution, were added and the sections were incubated at 4 °C overnight in a humid chamber. The sections were washed in PBS then incubated with secondary antibodies, diluted in blocking solution, for 1.5 h at room temperature. Sections were washed in PBS before adding DAPI (VWR) for < 1 min. Sections were mounted with Fluoromount (Southern Biotech). Fluorescent images were captured using Leica SP8 confocal microscope system.

For retinal immunofluorescence: Dissected eyes were first incubated with a blocking solution with 10% Serum and 0.05% Triton-X in PBS before overnight incubation with biotinylated *Griffonia simplicifolia* isolectin B4 mixed at a 1:500 dilution in blocking solution. Samples were washed three times for 5 min with PBS before incubation with streptavidin with fluorescent tag (Alexa Fluor 647—Thermofisher) in blocking solution at 1:1000 dilution for 1.5 h. Samples were washed again before incubation with Hoechst for 1 min. Samples were rinsed with PBS before retinal removal and mounting. Retinal flat-mounts were imaged with a Zeiss wide-field microscope, generating a tiled scan of the retina.

For human brain immunofluorescence: human deep white matter brain samples were non-neurological controls from the MRC-Edinburgh brain bank (16/ES/0084), chosen to have lack of or very minor pathology as confirmed by Prof. Colin Smith (Table 2). Sections (4 μm) of FFPE tissue were de-waxed and re-hydrated for 2 × 10 min in Xylene, 2 × 2 min in 100% ethanol, then 2 min in each of 95%, 80% and 70% of ethanol. Antigen retrieval was performed by microwaving the samples for 15 min in 0.01 M citrate buffer (pH = 6, Vector Laboratories). To quench the autofluorescence, slides were incubated for 1 min with Sudan Black, washed 2 × 5 min in TBS with 0.001% Triton, and incubated the slides in 3% H_2_O_2_ for 10 min. Afterwards, the slides were washed again and a serum block added (TBS, 0.5% v/v Triton, 10% v/v HIHS) for 1 h at room temperature. Antibodies were diluted in serum block and added to slides overnight at 4* °C*. Then, slides were washed 2 × 5 min in TBS + 0.001% v/v Triton and incubated with an ImmPRESS vector secondary antibody for 1 h. After another wash, Tyramide (Cy3) at 1:500 was added for 10 min, washed off and the slides boiled in 0.01 M citrate buffer (pH = 6) for 2.5 min and left in the hot buffer for 20 min. Slides were washed (2 × 5 min) and incubated overnight at 4* °C* with the second primary antibody diluted in serum block. On the third day, slides were washed (2 × 5 min), incubated with appropriate Vector ImmPRESS antibody (Vector Laboratories) for 1 h at room temperature, washed again (2 × 5 min) and incubated with Tyramide (Cy5, 1:500) for 10 min. After washing the slides (2 × 5 min) they were counterstained with Hoechst (1 μg/ml 10 min), washed (2 × 5 min) and mounted.

### Electron microscopy

Procedures were carried out blind to genotypes. Rats were transcardially perfused; first with PBS (< 2 min), then with fixative (4% paraformaldehyde w/v, Sigma; 0.1–2% glutaraldehyde, v/v, Electron Microscopy Sciences (EMS) or TAAB Laboratories Equipment Ltd.; in 0.1 M phosphate buffer, Sigma; 10 min). The brains were extracted, post-fixed in the same fixative (24 h at 4 °C), and vibratome-sectioned (50 μm thick).

For standard EM, sections were post-fixed with 1% osmium tetroxide (EMS; in 0.1 M PB; 30 min) then dehydrated in an ascending series of ethanol dilutions (Sigma; 50%, 70% (with 1% uranyl acetate; w/v; EMS), 95% and 100%) and acetone (EMS). Sections were then lifted into resin (Durcupan ACM; EMS), left overnight at room temperature, then placed on microscope slides, covered with coverslips, and resin cured at 65 °C for 3 days. Subcortical white matter (internal capsule) was imaged with brightfield light microscopy then cut from the slides, mounted on plastic blocks (Ted Pella) and ultrathin sectioned (70 nm thick) for EM. Serial sections were collected on to single-slot, formvar-coated copper grids (EMS), contrasted with lead citrate (EMS) and Uranyless (EMS) and imaged with the electron microscope (JOEL TEM-1400 plus). Magnifications were chosen to visualise each vessel with surrounding perivascular region for assessment.

For immuno-EM, sections were washed into cryoprotectant (10% glycerol v/v, Sigma; 25% sucrose w/v, Sigma; in 0.05 M PB, Sigma), incubated for 2 h (room temperature) then freeze-thawed 3 times with liquid nitrogen, and washed in cryoprotectant and PBS. Sections were then blocked with washes in 0.5% acetylated bovine serum albumin (BSAc; Sigma; in 0.1 M PB). Primary antibody (rabbit polyclonal ATP11B; Proteintech; 13,672–1-AP; 1:100 in 0.1% BSAc in PBS) was added to each section and incubated overnight, shaking at room temperature. Sections were washed out of primary antibody with 0.1% BSAc–PBS washes then secondary gold-conjugated antibody (Nanogold Fab' goat anti rabbit IgG; Nanoprobes; #2004; 1:100 in 0.1% BSAc–PBS) was added and incubated for 2 h shaking at room temperature. Sections were washed in PBS then sodium acetate trihydrate in preparation for silver intensification of gold particles, then reacted for 2.5 min each in silver reagent (HQ silver kit; Nanoprobes; #2012) before further washes in sodium acetate trihydrate then PBS. Sections were then processed as for standard EM (except osmium tetroxide post-fixation for only 8 min), and tissue examined with the electron microscope for ATP11B localisation.

### Tracer experiment

4-week-old WT and KO rats were lightly anaesthetised to allow tail vein catheter placement. Animals received a solution of fluorescently conjugated tracers (fluorescein isothiocyanate (FITC) wheat lectin from *Triticum vulgaris* (Sigma-Aldrich), tetramethylrhodamine (TMR) dextran (65–80 kDa) (Sigma-Aldrich) and cadaverine-647 (Thermofisher)) into the tail vein. For a positive control a rat received a tail vein injection of mannitol (7.7 ml/kg 20%, Fresenius Kabi Canada) just prior to tracer injection. Negative control animal was given tracer containing only FITC–wheat lectin from *Triticum vulgaris*. Animals were allowed to recover before euthanasia via intraperitoneal injection of Euthatal (150 mg/kg; Merial) 1 h after tracer injection. Brains were removed and fresh frozen before sections were cut at 10 μm thickness using a cryostat. Slices were collected in series so that each slide had a spread of coronal sections of the deep white matter. Slides were mounted using Vectashield Vibrance (Vector Laboratories).

### Image processing and quantification

For image analysis, an ImageJ macro was used to randomise image titles for blinded manual counting.

For brain images: Cell counting was carried out manually using max projections of z-stacks of 10 μm tissue at 20× generated with ImageJ and the Cell Counter plugin. For cultured EC CLDN5 quantification, cells were classified as CLDN5+ if the border with all neighbouring cells (delineated with ZO-1 staining) had CLDN5 present.

For EM micrograph: For vessel scores, each blood vessel was scored independently by 4 blinded researchers using a subjective scoring system of 0 (normal), 1 (mild) and 2 (severe abnormality) taking into account undulations of the luminal membrane, thickness of the EC, thickness of the basement membrane, components such as blood cells and membrane in the lumen as described in Suppl. Figure 1a,b, (online resource). Data were then illustrated as mean per animal and the modal score for each blood vessel to show heterogeneity. For the white matter changes by EM, the proportion of myelinated to unmyelinated axons in a 5 μm ring around each vessel was assessed. The percentage of disrupted tissue (vacuoles and myelin debris) compared to total area was measured within a 10 μm ring for each vessel using ImageJ and TrakEM2 plugin, and correlated with EC score (Suppl. Figure 1c,d, online resource). As with vessel scores, researchers were blinded to whether vessels came from WT or KO animals when measurements were made. Axon diameters were calculated by tracing the circumference of > 700 myelinated and > 350 unmyelinated axons, surrounding 70 vessels (31 WT, 39 KO) from 6 adult WT and 8 adult KO animals and deriving the diameter assuming a circle. For tracing and 3D reconstruction of ATP11B immunogold-EM samples, serial section micrographs of one vessel from a wildtype animal were aligned, and ECs traced and reconstructed with ImageJ and the TrakEM2 plugin. Background levels of immune-gold labelling were classed as that present over the nuclei of cells, equating to 2.5 particles/ μm^3^. Other vessels from this sample and a KO animal sample were also captured for assessment of ATP11B localisation.

For retinal images: In the plexus, regions of interest (ROIs) were selected in ImageJ using the diameter of the optic disc as a relative measurement. ROIs were taken from both proximal and distal selections in each of the four cut sections of the retina. ROIs then underwent segmentation in FIJI to generate a binary representation of the vasculature. ROIs were inverted, contrast enhanced and segmented using Phansalkar auto local thresholding to binarise the image [40], images were then filtered [9] and staining abnormalities accounted for [31]. Binarised ROIs then underwent skeletonisation via Voronoi tessellation as previously described [8]. Radius data for the skeleton was measured during skeletonisation by defining pixels on the boundary of the binary mask and measuring the distance erased to produce skeletons. Vessel tortuosity and branching were measured using a Python script which defines branching points as vertices in the skeleton with greater than 2 neighbouring vertices. This allows quantification of tortuosity by measuring actual versus Euclidean distances between these points. Branching index was calculated as the number of branching points per µm^2^. Major vessels were split into arterioles and venules for analysis and average vessel diameter, tortuosity and branching indices measured manually in ImageJ. Average vessel diameter was measured by taking manual measurements across the vessel at 200 µm intervals from vessel origin to terminal bifurcation. Code available at link below.

For PLP pixel count analysis: Vessels were manually delineated using IsoB4 staining from max projections of z-stacks of 10 μm tissue at 20 × generated with ImageJ, and a concentric ring (10 µm width) automatically generated around each vessel, adjusted to ensure that no two areas are analysed twice; a part of an outermost ring was subtracted if it overlapped with another vessel’s innermost ring. Intensity thresholds were set for each image to minimise background staining before a PLP channel pixel count was obtained for each ring, adjusting for ring area and depth of tissue section. Code available at link below.

For tracer experiment analysis: Images were acquired using a 40× oil lens and were deconvoluted using Huygens Suite (Scientific Volume Imaging) before analysis with ImageJ. A custom macro was written which measured the intensity along a line of set length for each of the 3 tracers. Longitudinal vessels were selected for the analysis and the lines drawn perpendicular to the vessel wall. The information was then exported and the FITC–lectin used to delineate the vessel. The average intensity of the cadaverine-647 and TMR-dextran was calculated outside the vessel. A minimum of 7 vessels per animal over 300 µm of brain in the corpus callosum were analysed.

For coverage of pericyte and astrocyte endfeet: Using a custom macro in ImageJ, vessels were automatically delineated and area calculated using IsoB4 staining from max projections of z-stacks of 10 μm tissue at 20x. This created an outline for each vessel. The macro overlays a separate channel that shows staining for another marker and calculates the pixel count for that marker withing the outline of each vessel. This generates a coverage value for each vessel. A mean of this value was calculated for each image taken. Five images per animal was used to calculate an overall mean coverage value per animal.

### Statistical analysis

Statistical analysis methods are described within the figure legends for each graph, all of which show mean ± SD. Graphs and statistical calculations were made using GraphPad Prism version 9.0.0/9.2.0 for Windows, GraphPad Software, San Diego, California USA, www.graphpad.com or RStudio Team (2021) (RStudio: Integrated Development Environment for R. RStudio, PBC, Boston, MA, www.rstudio.com). When comparing between two groups, (typically WT and KO), *t* tests were used (parametric data) and Mann–Whitney *U* (non-parametric). When comparing interactions of age or percentage tissue volume as well as genotype, ANOVA was used. For analysis of ventriculomegaly a Fisher’s exact test was performed. Statistical analysis of ventriculomegaly proportions within a genotype longitudinally was performed using a Wilcoxon signed rank test. For variance analysis a F test was performed and Pearson R for correlation. Significance codes were generated by GraphPad to report three significant digits. *p* < 0.05 was set as the criterium for statistical significance for all tests. *p* values less than 0.01 are summarised with 2 asterisks, < 0.001 with three asterisks, < 0.0001 with four asterisks.

### Biological/technical replicates

Unless otherwise stated, for all graphs for immunofluorescence staining quantification each point represents a different animal/cell culture (biological replicate), giving an average value calculated from a minimum of 5 fluorescence images (technical replicates). For all graphs for western blot densitometry analysis, each point represents a different cell culture lysate (biological replicate), an average value calculated from three western blot experiments (technical replicates). For behavioural experiments, each point represents a different animal (biological replicate).

## Results

### ATP11B is expressed by many brain cells in vivo in a heterogeneous pattern

**Fig. 1:**
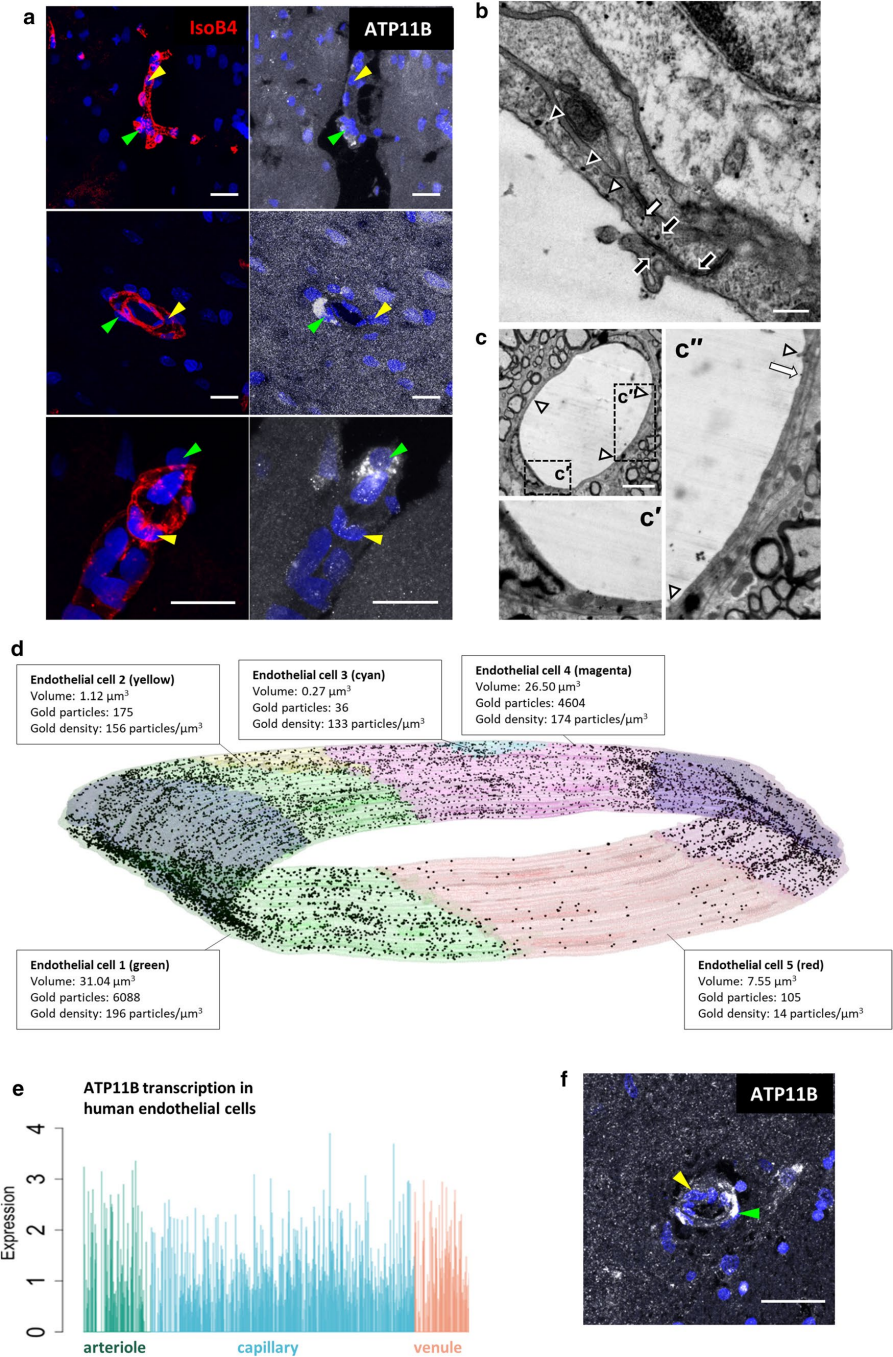
Atp11b/ATP11B is expressed heterogeneously in brain ECs. **a** Heterogeneous expression of ATP11B (white) in wildtype rat brain ECs of vessels (IsoB4 + , red); yellow arrows indicate no ATP11B expression, green arrows indicate ATP11B expression. DAPI nuclear stain—blue. Scale bars = 10 µm. **b** Localisation of ATP11B in wildtype rat brain ECs by immuno-gold electron microscopy to intracellular vesicles (black arrowhead), luminal membrane (white arrowhead), abluminal membrane blebs (white arrow) and either side and within a tight junction between two ECs (black arrows). Gold particles are also seen in adjacent astrocytic endfeet. Scale bar = 0.2 µm. **c** Varied expression of ATP11B immunogold labelling in neighbouring wildtype ECs in the same transverse section of a blood vessel. In a vessel formed of three ECs, joined by tight junctions (white arrowheads), one EC contains many ATP11B immunogold particles (**c’**), whereas the adjacent EC (**c’’**) contains only one (white arrow). Scale bar = 4 µm. **d** Heterogeneous expression of ATP11B (immunogold particles) in ECs of the same vessel in a wildtype animal seen in a 3D reconstruction of 5 EC profiles from 46 serial EM sections (70 nm-thick) (see also Suppl. Video 1, online resource). The density of gold particles per volume of EC profile (particles/µm^3^) shows that one of the five ECs (EC5, red) has a far lower density of ATP11B-gold than the other four ECs (ECs 1–4; green, yellow, cyan, magenta) by a factor of between 9.5 and 14, compared to the level of background gold labelling seen in the nuclei (blue) of ECs 1 and 4 (green/magenta); 2.5 particles/µm^3^). **e** Single nucleus heterogeneous expression of *ATP11B* transcript from snRNAseq in human adult CNS ECs, categorised into arteriole, capillary and venules [48]. **f** Heterogeneous expression of ATP11B (white) in human brain ECs of a vessel, yellow arrows indicate no ATP11B expression, green arrows indicate ATP11B expression. DAPI nuclear stain—blue. Scale bar = 10 µm

*Atp11b/ATP11B* is expressed by many brain cells, including ECs, as found in publicly available transcriptome databases from both rat and human brain (https://www.proteinatlas.org/, https://portal.brain-map.org/). We, therefore, investigated ATP11B expression at the protein level by immunofluorescence, using a validated ATP11B antibody, (Suppl. Figure 2, online resource) in the brain deep white matter of wildtype (WT) Sprague–Dawley rats. ECs, oligodendrocytes, pericytes, microglia, astrocytes and neurons all expressed ATP11B but in a heterogeneous way (Fig. 1a and Suppl. Figure 3, online resource). However, here, we focused on ECs as our cell of interest, due to our previous work showing EC dysfunction in the SHRSP and that knock-down/out of ATP11B expression in human and mouse ECs leads to EC dysfunction sufficient to stall oligodendrocyte differentiation [44]. In the same section of blood vessel, ATP11B-positive ECs are found adjacent to ATP11B-negative ECs (Fig. 1a–d), and ATP11B-positive cells account for 5–10% of all ECs, which does not change with age of WT rat (juveniles 6.52 ± 1.68%, adults 8.78 ± 2.07% (mean ± s.d.)). Furthermore, ATP11B-positive but heterogeneous immunostaining in ECs was present throughout the brain, in both grey and white matter (Suppl. Figure 3b, online resource).

To further confirm this heterogeneity and to identify the subcellular location of ATP11B, we used immunogold electron microscopy (EM) in the same deep white matter areas. ATP11B is involved in both membrane function and vesicular transport, and we identified gold particles indicating ATP11B in the EC cytoplasm, at the EC membrane surface and both at and within the tight junctions between ECs (Fig. 1b). This is consistent with the subcellular location of ATP11B in cell lines in vitro [38] and is the first localisation of ATP11B in ECs in the brain. The proximity of ATP11B labelling to EC tight junctions is of interest as we previously showed a reduction of tight junction protein CLDN5 in the SHRSP, where ATP11B is absent [44]. The immunogold EM also confirmed a clear heterogeneity in protein expression of ATP11B in ECs, where in a single blood vessel, seen in cross section in two dimensions, one EC expresses ATP11B, whereas the other two express no ATP11B (Fig. 1c). When we reconstructed a different blood vessel in three dimensions, and counted immunogold particles per volume as a measure of the amount of ATP11B expression, we confirmed the heterogeneity of expression, with one EC (red) with a very low number of gold particles compared to the others (Fig. 1d. and Suppl. Video 1, online resource). The EM analysis also showed no association between ATP11B-positive or negative ECs and surrounding pericytes or astrocyte endfeet (Suppl. Figure 3c,d, online resource).

Heterogeneous expression of ATP11B in ECs does not relate to vessel type, as this occurs in the same vessel section, and all vessels studied are very small (by definition) and capillaries. We capitalised on our Human Cell Atlas single nuclei RNAsequencing data in adult human brain tissue [48] subsetting for ECs, categorising them of arteriole, capillary and venule origin using scSorter [21], and visualising *ATP11B* expression on an individual cell basis. Here, we found clear heterogeneity of *ATP11B* RNA expression in human brain small vessel ECs (Fig. 1e), confirmed again at the protein level in human brain tissue (Fig. 1f). This heterogeneity was particularly intriguing as SVD pathology classically occurs in a patchy manner—around some small vessels but not others in the same deep white matter region [27]. We investigated the expression of ATP11B in ECs extracted and cultured from rat brain, and found that virtually all ECs expressed ATP11B (99.9% ± 0.127, mean ± s.d.), suggesting both strong in vivo and in vitro differences, and that cultured ECs would be a good technique to understand the function of ATP11B + ECs. Due to these findings, our previous work implicating ECs in SVD pathology and our wish to investigate pathology in the context of normotension, we decided to generate a ATP11BKO transgenic rat.

### *Atp11b*KO rat generation

**Fig. 2:**
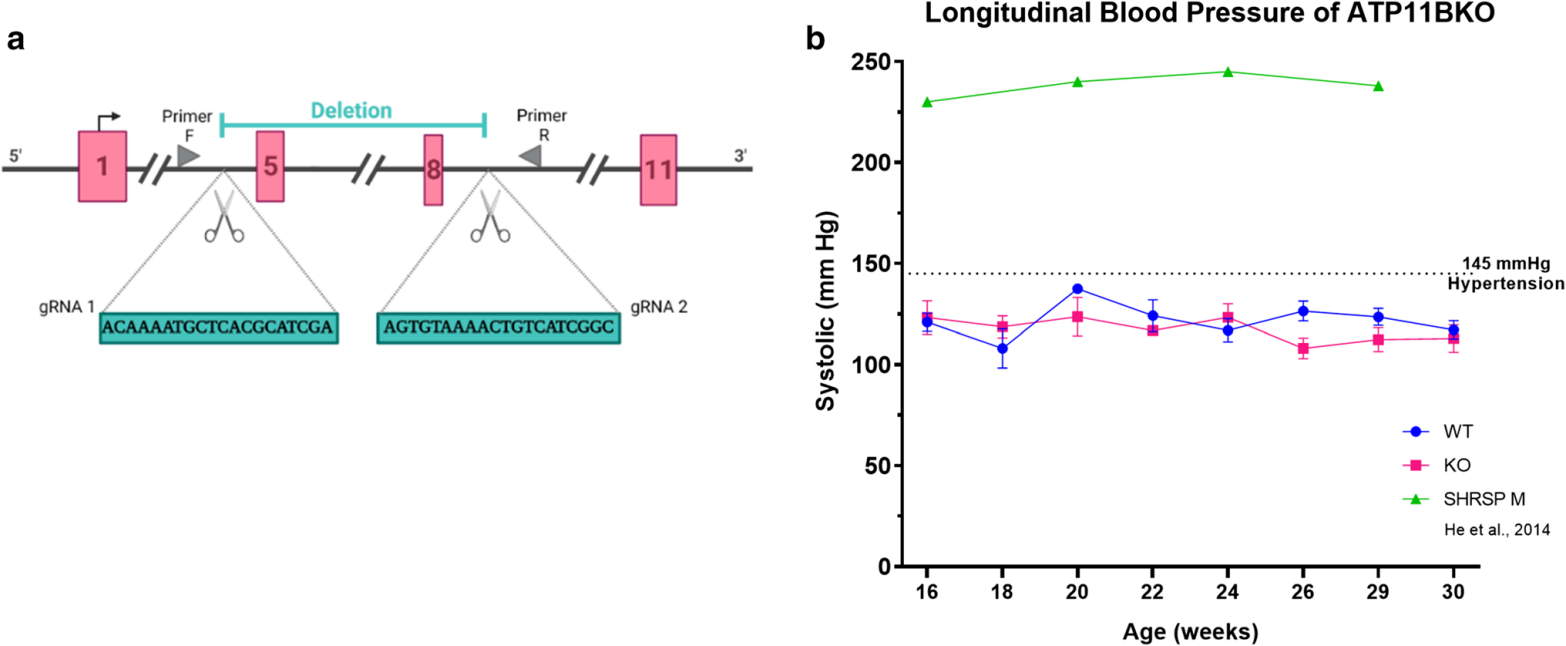
*Atp11b KO* rat. **a** *Atp11b*KO CRISPRCas9 deletion strategy. gRNAs induce non-homologous end joining (NHEJ) leading to deletion between exons 5 and 8, with knockout confirmed by PCR using primers in locations indicated. **b** Longitudinal blood pressure study on two groups of mixed-sex KO (11 animals) and age-matched controls (15 animals) shows that KO animals are normotensive (> 145 mmHg denoted as hypertension), and do not diverge from WT, unlike hypertensive male SHRSPs (data from [25]). Mean ± SD for whole group, using the average systolic reading from each animal

The SHRSP has a deletion mutation in *Atp11b* which predicts a truncated ATP11B protein, but leads to its total loss by western blot with an N-terminal antibody[44]. With the company Cyagen, we reproduced this deletion mutation in a new transgenic rat model on a Sprague–Dawley background, using CRISPR–Cas9 technology, with guide RNAs which disrupt from exon 5 to exon 8 (Fig. 2a and methods). Loss of ATP11B protein was confirmed with an ATP11B antibody by immunofluorescence and immunoEM (Suppl. Figure 2, online resource). These rats breed normally with no difference in litter frequency or stillbirths compared to WT (Suppl. Figure 4a, online resource) and reached 1 year of age without overt health problems. Body weights showed no difference between KO and WT juvenile animals (Males WT 67.7 ± 6.2 g KO 69 ± 5.3 g, Females WT 65.3 + /7.4, KO 61.3 ± 0.6 g) but there was a decrease in body weight in the adult KO males (Male WT 579.6 ± 27.4 g, KO 521.8 ± 31.6, Females WT 293.7 ± 9.3, KO 305.2 ± 5.8 g) (Suppl. Figure 4b, online resource), in line with that seen in the older SHRSP [18].

### The *Atp11b*KO rat is normotensive

Our strategy of selecting *Atp11b*, in which there is a mutation in the SHRSP (a rat with SVD pathology) but which is not found in the SHR (a rat with hypertension but lacking overt SVD pathology) led to our prediction that the *Atp11b*KO rat would be normotensive. In a longitudinal study, there indeed was no difference in blood pressure of the *Atp11b*KO rat compared to WT controls at any age, with the average reading not surpassing 145 mmHg in either group, denoted as hypertension in Sprague–Dawley rats [11] (Fig. 2b), and no difference between males and females. They are clearly different to the hypertensive SHRSP model rats at the same ages [25].

Next, we investigated whether, without hypertension and without ATP11B, there were still signs of EC dysfunction at a juvenile age (3–6 weeks) and an adult age (24–30 weeks).

### *Atp11b*KO-ECs show molecular signatures of dysfunction despite the absence of hypertension

**Fig. 3:**
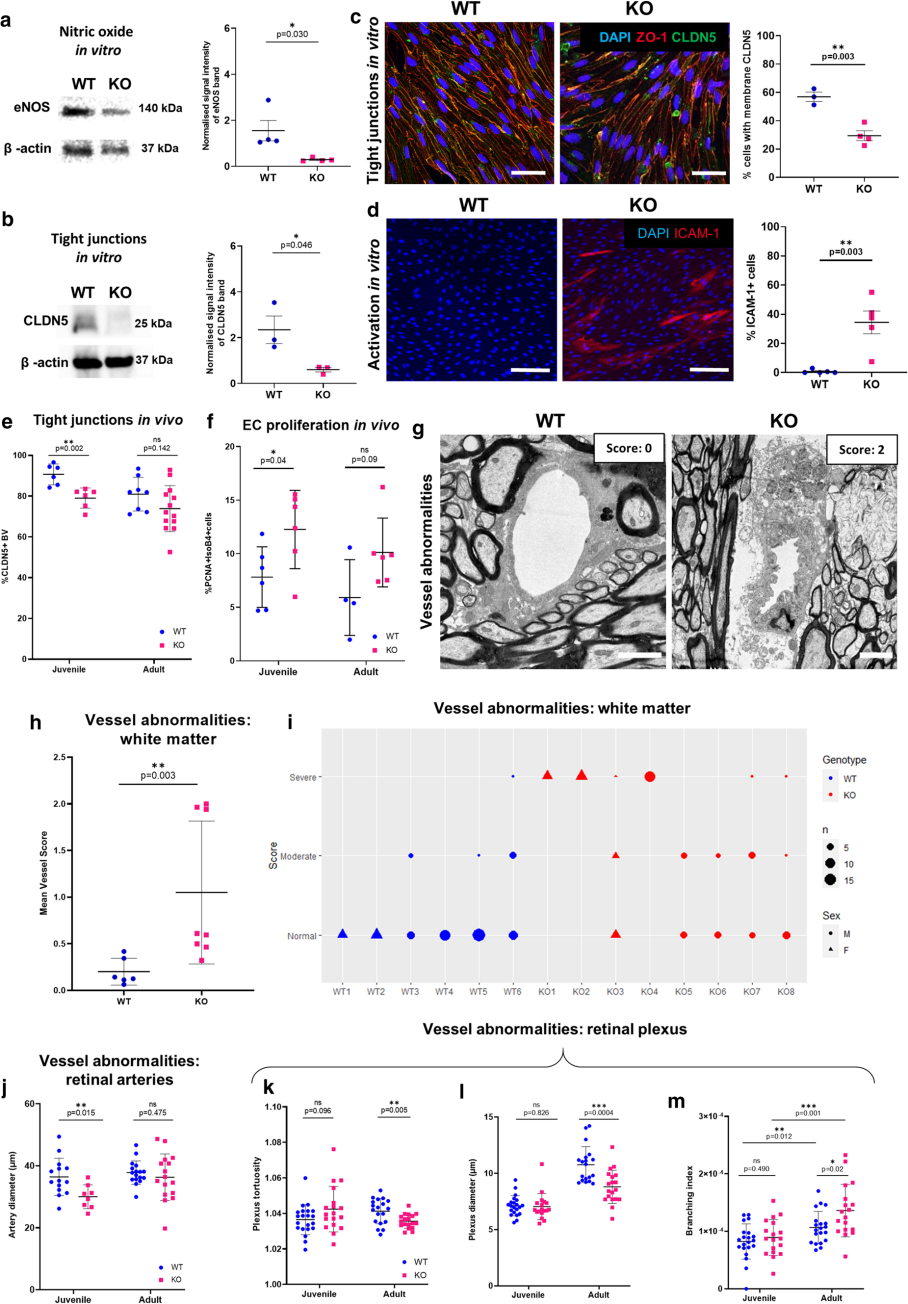
*Atp11b*KO-ECs are inherently dysfunctional. KO-ECs in vitro show dysfunction with expression of significantly less **a** eNOS (*t*-test, *p *= 0.030, *t* = 2.825, *df *= 6) and **b** CLDN5 (*t*-test, *p* = 0.046, *t *= 2.826, *df *= 4) than WT-ECs: western blot of cell lysates, with quantification by densitometry (relative to β-actin), calculated from images taken with LiCor image system for eNOS and from photographic film for CLDN5. **c** Fewer cultured KO-ECs express the tight junction marker CLDN5 (green) compared to WT-ECs at the border between all neighbouring cells, delineated by ZO-1 (red), (DAPI-stained nuclei—blue) with quantification of % cells with membranous CLDN5, (*t*-test, *p* = 0.003, *t* = 5.553, *df* = 5) scale bar = 20 µm. **d** More cultured KO-ECs express the endothelial activation marker ICAM-1 compared to WT-ECs, with quantification (*t*-test, *p* = 0.003, *t* = 4.306 *df* = 8), *p* = 0.003, *t* = 4.306 *df* = 8). **e** Fewer tight junction CLDN5 + blood vessels in *Atp11b*KO tissue from juvenile age group compared to WT (*t*-test, *p* = 0.002, *t* = 4.018, *df* = 10). **f** More proliferating ECs (PCNA + IsoB4 +) in *Atp11b*KO tissue from juvenile age group compared to WT (*t*-test, *p* = 0.040, *t* = 2.357, *df* = 10). **g** Marked abnormalities in adult KO blood vessels shown with electron microscopy (scale bars 2 µm). **h** Abnormalities in ECs quantified as mean vessel score per animal (Mann–Whitney *U* = 2, *p* = 0.003), full descriptors used to score are found in Suppl. Figure 1, online resource and methods. **i** Heterogeneity is seen between vessels within animals; plotted as modal rater scores for each vessel analysed. **j** Retinal artery diameter is significantly smaller in juvenile KO animals compared with WT animals (*t*-test, *p* = 0.015, *t* = 2.688, *df *= 20) but not at the adult age (*t*-test *p* = 0.475, *t* = 0.724, *df* = 31). Adult KO retinal plexus vessels show **k** significantly less tortuosity (*t*-test, *p* = 0.005, *t* = 2.969, *df *= 35), **l** significantly smaller diameter (*t*-test, *p* = 0.0004, *t *= 3.934, *df* = 36), and **m** significantly increased branching (*t*-test, *p* = 0.020, *t* = 2.430, *df* = 36), with increased branching with age in both groups: KO animals (*t*-test, *p* = 0.001, *t* = 3.584, *df* = 34), WT animals (*t*-test, *p *= 0.012, *t *= 2.641, *df *= 39). There are no identified differences between plexus measurements by genotypes at the juvenile age (*t*-tests, Diameter: *p *= 0.826, *t* = 0.221, *df* = 37; Branching: *p *= 0.490, *t *= 0.6974, *df *= 37; Tortuosity *p* = 0.096, *t* = 1.710, *df *= 37). All graphs show mean ± SD

EC dysfunction can be defined by several molecular signatures that parallel the microvascular dysfunctions observed macroscopically in human SVD, including loss of eNOS indicating less NO production, reduction in CLDN5 tight junction protein indicating loss of BBB integrity, and an upregulation in activation markers, such as Intercellular Adhesion Molecule 1 (ICAM-1) [6, 24, 62]. ECs cultured from the brains of neonatal *Atp11b*KO rats (KO-ECs) compared to WT-ECs show a significant reduction in the amount of eNOS (Fig. 3a), a significant reduction in amount of CLDN5 and a change in its location, as defined by western blot and immunofluorescence, respectively (Fig. 3b, c) and a significant upregulation of ICAM-1 (Fig. 3d). This suggests inherent EC dysfunction similar to that seen in cultured SHRSP ECs and in 3-week-old SHRSPs [44].

In the deep frontal white matter of rat brain at juvenile ages, we found significantly fewer CLDN5+ blood vessels (Isolectin(Iso)B4 +) and increased proliferation of ECs (PCNA+ IsoB4+) overall in *Atp11b*KO juvenile rats (Fig. 3e, f). However, as in the young SHRSP [44] these markers of EC dysfunction did not disrupt BBB integrity to intravenous injected fluorescent tracers at this age (Suppl. Figure 5, online resource). The tight junction and proliferation marker results in adults were more variable, in-keeping with our demonstration of heterogeneous ATP11B expression in ECs in vivo in WT rats. Therefore, we turned to EM analysis to assess EC ultrastructural abnormalities in the same area in this adult age group. Four blinded independent researchers graded EC pathology as 0 (normal), 1 (mild) and 2 (severe abnormality), finding increased EC inner surface undulations, luminal blood cell stasis and abluminal thickened basement membrane in adult KO animals compared to controls (Fig. 3g–i, Suppl. Figure 1a,b, online resource). These changes were more frequent in KO animals (Fig. 3h, i), indicating EC dysfunction at an ultrastructural level, consistent with structural abnormalities seen in small perforating brain blood vessels in human SVD [27]. Not all blood vessels were affected, with variability between vessels even within animals (Fig. 3i), again consistent with the heterogeneity of expression of ATP11B we saw in the WT vessel ECs and highlighting the well-recognised focal nature of SVD as found in humans [27]. There was no difference in the coverage of pericytes and astrocyte end feet around blood vessels between WT and KO groups at either age (Suppl. Figure 6a,b, online resource).

In humans, retinal imaging is being explored as a non-invasive diagnostic tool for SVD in patients [34] as the retina can be considered as a ‘window’ to the brain, with shared embryological origin and neurodegenerative pathways, and as retinal vessels are relatively easily measurable in vivo. Therefore, we next examined the larger retinal arterioles/venules and smaller plexus vessels in flat-mount retinal preparations [13] labelled with IsoB4 in juvenile and adult animals. Reduced retinal artery (but not venule) diameter was observed within the KO animals in juveniles only (Fig. 3j, Suppl. Figure 7, online resource). Meanwhile, reduced diameter and tortuosity but increased branching of plexus vessels were noted exclusively in the adult animals (Fig. 3k–l). This may reflect early impaired retinal artery vasodilation (possibly secondary to NO loss) and a later compensatory response to hypoxia leading to increased plexus vessel branching via an angiogenic response, supported by the increase in branching index in adult KO plexus vessels compared to juveniles (Fig. 3m). Human fundus imaging studies show that reductions in retinal arteriolar diameter, density and fractal dimension are associated with SVD features on MRI, independent of vascular risk factors [15], and plexus vessels can be imaged using optical coherence tomography angiography. Although some of these human retinal features are also present with hypertension, we can be clear in these KO rats that these abnormalities are independent of hypertension. Thus, hypertension is not required to produce EC dysfunction and blood vessel changes in the *Atp11b*KO rat. Next, we investigated whether white matter changes were also present.

### The *Atp11b*KO rat has white matter changes similar to those in human sporadic SVD

**Fig. 4:**
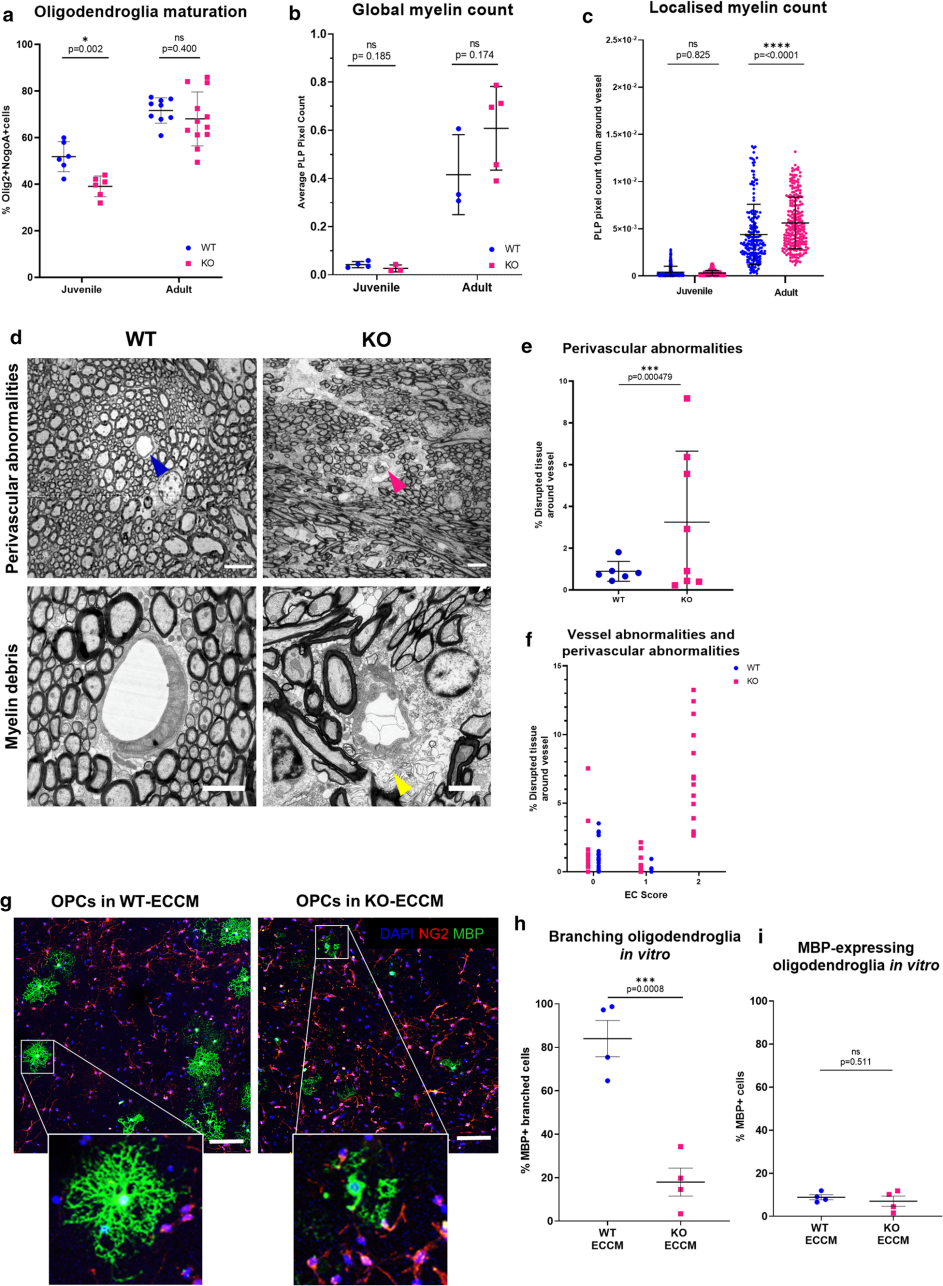
*Atp11b*KO white matter is abnormal. **a** Significantly fewer NogoA + oligodendroglia are present in the juvenile KO compared to WT animals (*t*-test, *p* = 0.002, *t *= 3.982, *df *= 10), but not at the adult age (*t-*test, *p* = 0.400, *t* = 0.8605, *df* = 19). **b** There are no gross myelin defects as assessed by PLP + pixel count in deep white matter of KO rats, as juveniles (*t*-test, *p* = 0.185, *t *= 1.536, *df *= 5) or adults (*t*-test, *p* = 0.174, *t* = 1.540, *df* = 6) (each point represents the average pixel count from six images of the deep white matter of one animal). **c** In adult KO animals, more PLP + labelling is present in a 10 µm wide ring adjacent to small vessels, compared to WT rats (One-way ANOVA, Tukey’s multiple comparison test, Juvenile: adjusted *p* = 0.825, *q* = 1.217, *df *= 1314, Adult: adjusted *p* =  < 0.0001, *q *= 10.28, df = 1314) (each point represents the pixel count for a single blood vessel, accounting for area and adjusted across experiments for the overall PLP pixel count for each image). **d** EM examples of perivascular changes in KO animals compared to WT (blue and pink arrowheads indicate blood vessels) at lower magnification (scale bar = 5 µm) and at higher magnification (scale bar = 2 µm) with yellow arrowhead indicating myelin debris. **e** KO animals display heterogeneity in white matter tissue disruption. There is a significant difference in the variance of the percentage disrupted tissue between WT and KO in a 10 µm radius from the vessel (*F* test, *F* = 50, *p* = 0.000479). **f** Heterogeneity in tissue disruption is seen within animals and between vessels. There is a significant positive correlation (Pearson *r*, *r *= 0.658, *p* =  < 0.0001) between vessel EC abnormalities and surrounding tissue disruption. **g–i** Oligodendrocyte precursor cells (OPCs) grown in EC conditioned media (ECCM) from cultures of KO-ECs show less branched morphology of MBP + cells, compared to OPCs grown in ECCM from cultures of WT-ECs, as shown by immunostaining for oligodendroglial markers NG2 (red) and MBP (green), (scale bar 20 µm) and percentage of branched MBP + cells **h** (*t*-test, *p* = 0.0008, *t* = 6.252, *df* = 6) but no difference in the number of MBP + cells **i**, (*t*-test, *p* = 0.511, *t* = 0.6987, *df* = 6). All graphs show mean ± SD

In the *Atp11b*KO brain deep frontal white matter (WM), there were significantly fewer mature Olig2 + NogoA + oligodendroglia in the deep frontal white matter in the juveniles, suggesting a WM disturbance, but not in the adults (Fig. 4a). This shows a similar pattern to the reduction in CLDN5 expression in small blood vessels in the same area of juvenile brains only (Fig. 3e), suggesting that EC-secreted factors change in how they affect oligodendroglia over time, with compensation. However, there were no gross general myelin defects, as assessed by Proteolipid protein (PLP) immunofluorescence, and there was the expected increase in amount of myelination between juveniles and adults as most myelination occurs postnatally (Fig. 4b). In human SVD, subtle diffuse WM changes are seen by MRI [7, 35], but pathological white matter changes are usually described as focal around blood vessels [3] and so we quantified the PLP-positive immunofluorescence pixels in a 10 µm concentric ring around small vessels of the deep white matter, with the hypothesis that there would be less myelin nearer to blood vessels. Instead, there was a significant increase in the number of PLP-positive pixels (a myelin marker) found near these vessels in the *Atp11b*KO adults (Fig. 4c), and we speculated that this was secondary to PLP-positive myelin debris in these areas. To test this, we returned to EM to investigate the ultrastructure of the myelin in adult animals. There was no difference in the proportion of myelinated to unmyelinated axons in a 5 µm ring around the vessels either when assessed as the mean per animal (myelinated axons mean ± s.e.m. 70.9 ± 3.1% WT, 72.7 ± 2.1% KO) or around each individual vessel. However, there was increased tissue disruption as measured by the percentage of myelin debris and vacuoles within a 10 µm ring around the vessels in KO animals, but with vessel-to-vessel variability (Fig. 4e, f). There was also a significant positive correlation between the EC and the disrupted tissue scores, so that those ECs with severe abnormality (score = 2) had consistently higher perivascular disruption (Fig. 4f). Increased myelin debris may also result from reduced debris disposal, but there is no difference in the number of microglia between KO and WT groups in this area, suggesting this is less likely (Suppl. Figure 6c, online resource). This disruption is also not simply due to changes in the surrounding axons as there is no difference in the axonal densities as measured by EM between the adult KO and control groups (WT (*n* = 6): 0.75 axons/µm^2^ ± 0.09 (SEM), KO (*n* = 8): 0.69 axons/µm^2^ ± 0.14 (SEM)) and no difference in either myelinated or unmyelinated axon calibre as measured on EM images between the adult KO and control groups: myelinated (*t* test, *p* = 0.570, *t* = 0.575, *df *= 12), unmyelinated (*t* test, *p* = 0.966, *t* = 0.0442, *df* = 12) (Suppl. Figure 6e, online resource). This supports our hypothesis that variable EC changes (which may depend on rat EC heterogeneity of ATP11B expression (Fig. 1)) drive the perivascularly-localised white matter changes seen in this model, and indeed in human SVD.

To further assess whether this spatial and temporal correlation of EC dysfunction and oligodendroglia changes could be linked, we isolated these cell types from neonatal WT and KO animals for in vitro experiments, which have the advantage of being reductionist, and as we know that virtually all WT ECs express ATP11B (as above) allowing easier dissection of its effects. In the SHRSP, we previously showed that SHRSP ECs secrete more HSP90α than controls, leading to arrest of oligodendrocyte precursor cell (OPC) differentiation, reducing the number of mature myelin basic protein (MBP)-positive cells[44]. Here, in a similar experiment, using cultured *Atp11b*KO ECs to condition media that was added to WT OPCs, there was no difference in the number of mature MBP+ oligodendroglia, but instead these *Atp11b*KO ECCM-treated MBP+ oligodendroglia showed a marked change in cell morphology with few and short processes, compared to the complexity of branching in controls, indicative of a maturation block at a later stage (Fig. 4g–i). As cultured oligodendroglia also express *Atp11b* at the transcript [43] and at the protein level (Suppl. Figure 8a, online resource), we were also interested in whether loss of *Atp11b* may change oligodendroglial behaviour independent of the EC effect, but isolated OPCs from neonatal *Atp11b*KO rats showed no difference in their survival, proliferation (Ki67+) or differentiation (MBP+) compared to WT OPCs in vitro (Suppl. Figure 8b-e, online resource), indicating that the maturation block and morphological changes we see in vivo is likely secondary to the EC effect. Thus, we have evidence of white matter damage in a patchy nature around small vessels, related to endothelial dysfunction in the *Atp11b*KO rat despite lack of hypertension. To relate this further to human SVD, we next assessed the rats with MRI, the route for human SVD diagnosis.

### The *Atp11b*KO rat has MR changes similar to human SVD

**Fig. 5:**
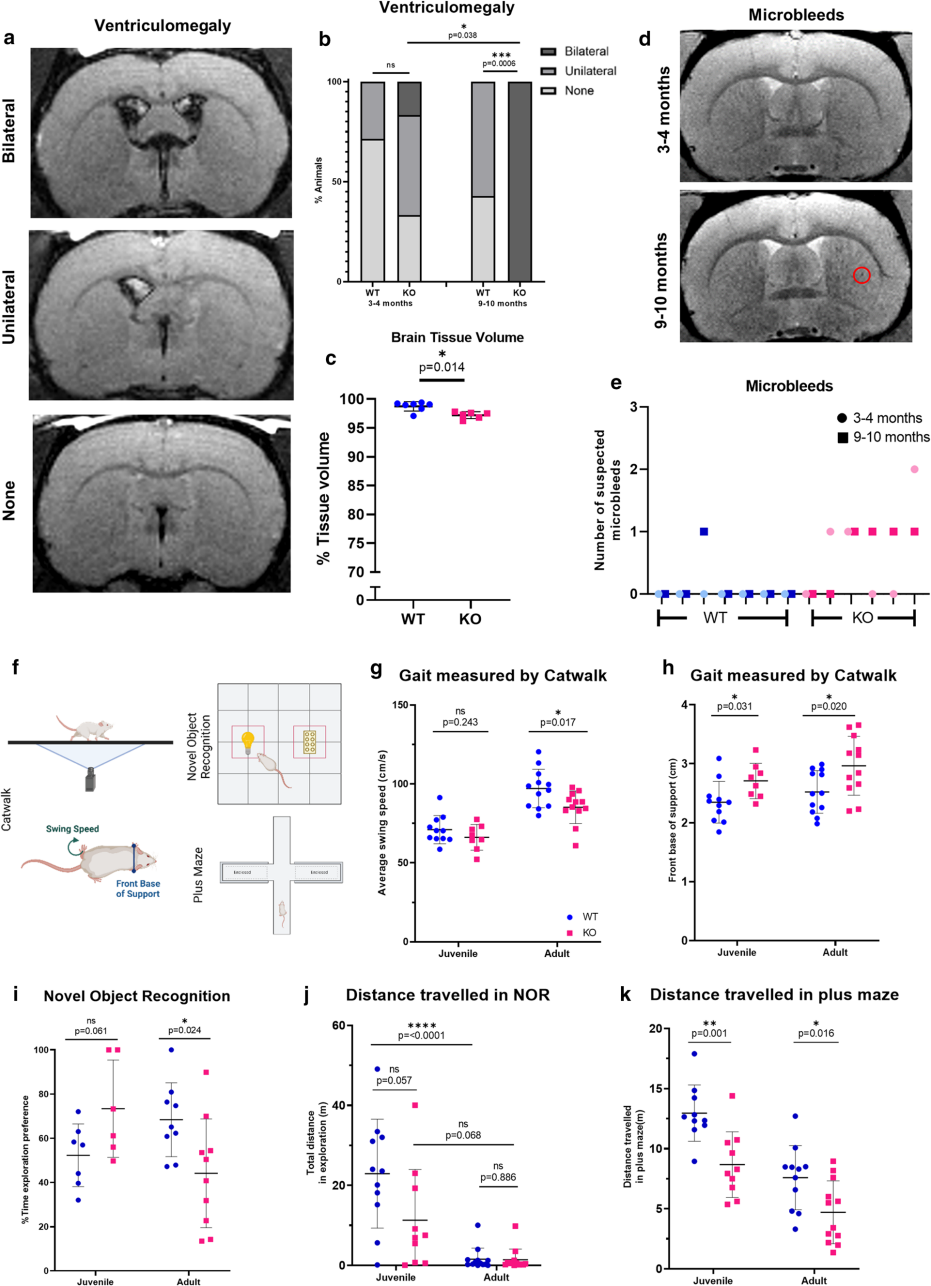
*Atp11b*KO rat has MR and behavioural changes similar to SVD. **a** Illustrative images of the lateral and third ventricles on fluid-attenuated inversion recovery (FLAIR) sequences showing (from top to bottom) bilateral ventricular enlargement, unilateral ventricular enlargement and normal ventricles. **b** Significant difference in ventriculomegaly at 9–10 months of age between WT and KO (Fisher’s exact test, *p* = 0.0006). Significant difference in the relative proportions of normal, unilateral and bilateral scores between 3–4-month and 9–10-month-old KO rat (Wilcoxon signed-rank test, *p* = 0.038) but not in the WT (Wilcoxon signed-rank test, *p* = 0.317). **c** Significant loss of brain tissue volume with bilateral ventriculomegaly (Mann Whitney *U* = 4, *p* = 0.014). **d** Illustrative images of same rat brain at 3–4 months which then develops a suspected microbleed at 9–10 months (red circle). **e** KO animals have more suspected microbleeds at both ages compared to WT. Each tick mark represents one animal imaged at 3–4 months and 9–10 months. **f** Diagram to show behavioural experiments carried out with juvenile and adult animals. Image made with BioRender.com. **g**, **h** KO animals have altered gait on Catwalk, with **g** reduced average swing speed of all paws at the adult age (*t*-test, *p* = 0.017, *t *= 2.572, *df* = 22) but not at the juvenile age (*t*-test, *p* = 0.243, *t* = 1.210, *df *= 17) and **h** increased front base of support at both juvenile (*t-*test, *p* = 0.031, *t *= 2.348, *df* = 17) and adult (*t*-test, *p* = 0.020, *t *= 2.499, *df* = 22) ages. **i**, **j** Memory deficits emerge in KO animals—novel object recognition **i**, where KO animals spend less time investigating the novel object compared to WT at the adult age (*t*-test, *p* = 0.024, *t* = 2.482, *df* = 17) suggesting memory failure, despite no difference in distance travelled compared to WT animals, at both ages **j** (*t*-tests, juvenile: *p* = 0.057, *t *= 2.024, *df *= 19; adult: *p* = 0.886, *t *= 0.145, *df *= 24). In addition, WT animals travel significantly less as they age (ANOVA, adjusted *p* =  < 0.0001, *q* = 7.421, *df* = 40), while KO animals show no significant difference **j**, (ANOVA, adjusted *p* = 0.068, *q* = 3.600, *df* = 40) suggesting early deficits. **k** KO animals travel less distance in the plus maze compared to WT at both juvenile and adult stage, potentially indicating apathy (t-tests, juvenile: *p* = 0.001, *t *= 3.762, *df* = 18; adult: *p* = 0.016, *t *= 2.607, *df *= 21)

A greater extent of human white matter changes as seen on MR correlates with a worse cognitive state [37] and allows SVD diagnosis. We performed longitudinal MR scanning using T1-weighted, T2-weighted, fluid attenuated inversion recovery (FLAIR) and T2*-weighted sequences, on a cohort of rats both at 3–4 months and 9–10 months and found clear differences to the WT controls, with all KO rats having ventriculomegaly at the later age (Fig. 5a, b). As expected, ventriculomegaly was associated with loss of tissue volume (Fig. 5c), suggestive of neurodegeneration and consistent with brain atrophy and ventricular enlargement in human SVD [20]. Spontaneous ventriculomegaly with age has previously been noted in adult Sprague Dawley rats [23, 32] but here it is accelerated in *Atp11b*KO rats. Furthermore, there was also MR evidence of microbleeds in the KOs at both ages (Fig. 5d,e), again consistent with human SVD [55]. The majority of apparent microbleeds in the older KOs were new, with only one lesion persisting from the younger age. There was no detectable change in white matter structural size measured at three locations in the corpus callosum, consistent with the focal white matter changes seen on pathology (Suppl. Figure 9, online resource). We next analysed how these MR and pathological changes affected rat behaviour.

### The *Atp11b*KO rat has behavioural changes similar to SVD

SVD patients can present with changes in altered gait, cognitive function and more subtle neuropsychiatric symptoms, such as increased apathy [12], so we chose behavioural tests to probe these parameters (Fig. 5f). Similarly to humans with SVD, KO rats have problems with gait, measured using the Catwalk equipment. *Atp11b*KO animals are slower (reduced swing speed—Fig. 5g) and more unsteady (increased base of support—Fig. 5h). They also have cognitive problems, with less interaction with novel objects in the Novel Object Recognition (NOR) test compared to WT age-matched controls in the adult age group, suggesting poorer memory that develops with age (Fig. 5i), despite normal movement in terms of distance travelled (Fig. 5j). Furthermore, in the elevated plus maze, KO rats at both ages travelled significantly less distance overall (Fig. 5k). This may reflect a combination of both mobility and cognitive issues, which are difficult to robustly distinguish. Rats move more in this test than Novel Object Recognition test, and so the relatively subtle mobility deficit in the KO rats may be revealed. However, KO rats move without problem for food and breeding, as shown by no difference in body weight of developing juvenile animals (as above) (Suppl. Figure 4b, online resource) and no difference in frequency of litters (Suppl. Figure 4a, online resource). Therefore, this finding may also suggest an impact from cognitive changes, perhaps with an additional degree of apathy or lack of motivation in the KO rats, which was also identified in the human study [12]. This is supported by a decrease in body weight in the older males (Male WT 579.6 ± 27.4 g, KO 521.8 ± 31.6, Females WT 293.7 ± 9.3, KO 305.2 ± 5.8 g) (Suppl. Figure 4b, online resource) and reflects what we saw in the SHRSP [18].

## Discussion

The *Atp11b*KO rat has EC dysfunction, white matter pathological and MR-identified changes, and a behavioural phenotype similar to human SVD in the absence of hypertension. This work also provides a hypothesis as to why this pathology is classically patchy, due to the heterogenous nature of ECs even in the same blood vessel, leading to variable dysfunction.

We show that the loss of *Atp11b* is sufficient to cause EC dysfunction, molecularly and structurally, which alters with age and disease progression, and leads to disordered communication with surrounding oligodendroglia, laying the ground to identify therapeutic targets to reverse these changes. ATP11B is a phospholipid flippase, thought to move the phospholipids phosphatidylserine (PS) and phosphatidylethanolamine (PE) from the outer surface of a membrane to the inner, either at the plasma membrane or at vesicle membranes involved in vesicular transport [38]. We show that in brain ECs, its location is consistent with both roles, found in the golgi, endoplasmic reticulum and plasmalemma. *Atp11b* deletion, through its alteration in phospholipid location in membranes is, therefore, likely to have many downstream consequences, including altering membrane dynamics, effects on vesicular transport and content, consistent with our conditioned medium experiments, tight junction expression (and thus BBB integrity) and membrane receptors and ion channels (potentially altering signalling). Furthermore, exposed plasmalemmal PS and PE are known ‘eat me’ signals for myeloid cells [36].

This is a global rat KO of *Atp11b*, and so even though the EC-effect seems marked and we have focused on this here, other cells may also be affected, with the potential for compensation by other flippases (e.g., ATP11A/C). The global *Atp11b*KO mouse shows impairment of hippocampal synaptic plasticity, but other phenotypes were not described, possibly secondary to less white matter in mouse [52], and conditional mutants or cell-selective rescue of ATP11B will be needed to tease out the relative contributions of each cell type to each pathology. Our previous identification of a SNP in *ATP11B* associated with human SVD MR changes (albeit with unknown function) and current finding of ATP11B expression in human brain indicate that this has human relevance and is worth further investigation. A link between EC dysfunction and OPC maturation arrest with myelin abnormalities is also found in the rare and familial SVD cathepsin A-related arteriopathy with strokes and leukoencephalopathy (CARASAL) [10], suggesting that there may be other molecular deficits affecting this crosstalk that are hitherto unrecognised causes of sporadic SVD.

Our findings overturn the classical view that hypertension is required for the changes in the SHRSP and in human sporadic SVD, and refocuses our attention on intrinsic endothelial dysfunction as the key mechanism underpinning this disease, with vessel to vessel variability, occurring early and inducing vulnerability of the white matter. Extrinsic environmental factors such as hypertension can also lead to EC dysfunction, but the intrinsic EC vulnerability in SVD-affected individuals may induce enhanced sensitivity to such later exposures and explains why it has proved difficult to prevent WMH progression even with intensive blood pressure reduction[56]. We believe that focusing on these mechanisms, rather than only on vascular risk factors, such as hypertension, will be fruitful, especially as it is increasingly recognised that vascular changes contribute to several dementia types, including FTD and AD [2, 45, 60]. New European SVD guidelines lament the lack of effective treatments for SVD [54] and clearly state that although hypertension can contribute to the disease and should be treated, other therapies are needed. Here, we show that there is now a real opportunity to target EC dysfunction and its crosstalk with oligodendroglia for treatment of dementia.

## Supplementary Information

Below is the link to the electronic supplementary material.

Supplementary file1 (PDF 6730 kb)

Supplementary Video 1. Heterogeneous expression of ATP11B in ECs of the same vessel. A 360° rotation of the 3D reconstruction shown in Fig. 1d, showing 5 EC profiles traced and reconstructed in 46 serial sections. Four of the ECs (ECs 1-4, green, yellow, cyan and magenta) show high densities of ATP11B-gold labelling (133-196 particles/ µm3), whereas EC5 (red) shows a much lower density of gold labelling (14 particles/ µm3), compared to the level of background gold labelling seen in the nuclei (blue) of ECs 1 and 4 (green/magenta); 2.5 particles/ µm3. For scale, see Fig. 1d. (AVI 25240 kb)

## Data Availability

Code available https://github.com/mobernabeu/normotensiveSVDrat for retinal vessel analysis and PLP pixel count, https://github.com/Anna-Williams/ATP11B_Quick_Procter_2022 for RNAseq.
