# Supplementary material for: Loss of the heterogeneous expression of flippase ATP11B leads to cerebral small vessel disease in a normotensive rat model
Source: Acta Neuropathol. 2022 May 30;144(2):283–303. doi: 10.1007/s00401-022-02441-4 (PMC9288385; doi:10.1007/s00401-022-02441-4)
Supplement: Supplementary file 1 — Supplementary file1 (PDF 6730 kb) [file 401_2022_2441_MOESM1_ESM.pdf]

Supplementary figures and legends

Loss of the heterogeneous expression of flippase ATP11B leads to cerebral Small Vessel Disease in a normotensive rat model

Authors: Sophie Quick[1#], Tessa V. Procter[1#], Jonathan Moss[1,3], Luise Seeker[1], Marc Walton[1], Angus Lawson[1], Serena Baker[1], Anna Beletski[1], Daniela Jaime Garcia[1], Mehreen Mohammad[3], William Mungall[5], Ami Onishi[5], Zuzanna Tobola[2], Michael Stringer[2], Maurits A. Jansen[6], Antoine Vallatos[2], Ylenia Giarratano[4], Miguel O. Bernabeu[4], Joanna M. Wardlaw[2,3], Anna Williams[1,3*].

# these authors contributed equally

Affiliations:

[1]Centre for Regenerative Medicine, Institute for Regeneration and Repair, University of Edinburgh; Edinburgh, EH16 4UU, UK.

[2]Centre for Clinical Brain Sciences, Edinburgh Imaging, Row Fogo Centre for Research into Ageing and the Brain, University of Edinburgh; Edinburgh, EH16 4SB, UK.

[3]UK Dementia Research Institute, University of Edinburgh; Edinburgh, EH16 4SB, UK.

[4]Usher Institute, College of Medicine and Veterinary Medicine, Bayes Centre, College of Science and Engineering, University of Edinburgh; Edinburgh, EH16 4UX, UK.

[5]Bioresearch and Veterinary Services, University of Edinburgh; Edinburgh, EH16 4SB, UK.

[6]Centre for Cardiovascular Science, University of Edinburgh; Edinburgh, EH16 4SB, UK

*Corresponding author. Email anna.williams@ed.ac.uk

a

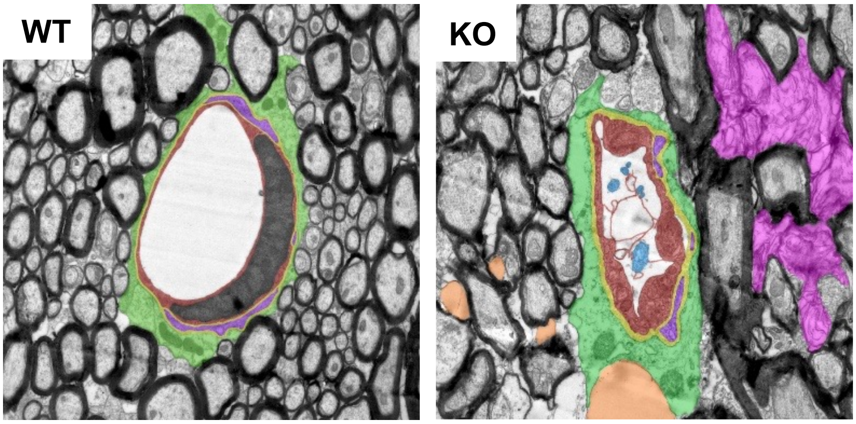

b

Descriptors for scoring
**Blood vessel scoring:** Undulations of luminal membrane (red)
Thickness of EC (red)
Thickness of basement membrane (yellow)
Components in lumen e.g. blood cells (blue) or membrane (red)

c

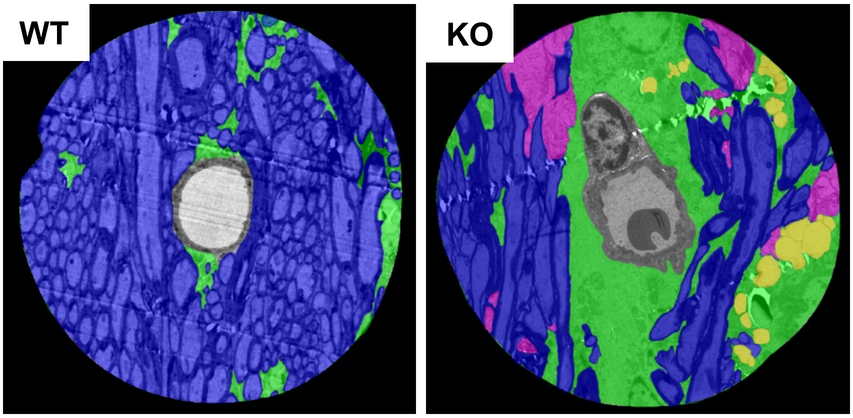

d

Descriptors for area calculations
Total area minus vessel (green + blue + pink + yellow)
Myelin (blue + pink)
Disrupted myelin (pink)
Vacuoles (yellow)

Supplementary Fig. 1 Electron microscopy grading system

a) Example of normal and abnormal endothelial cell images, with tables of descriptors (b). Additional features highlighted: astrocytes-green; pericytes-purple; EC nuclei-grey. (c) Example of measured areas of normal and abnormal tissue surrounding the vessel, with (d) table of descriptors.

a

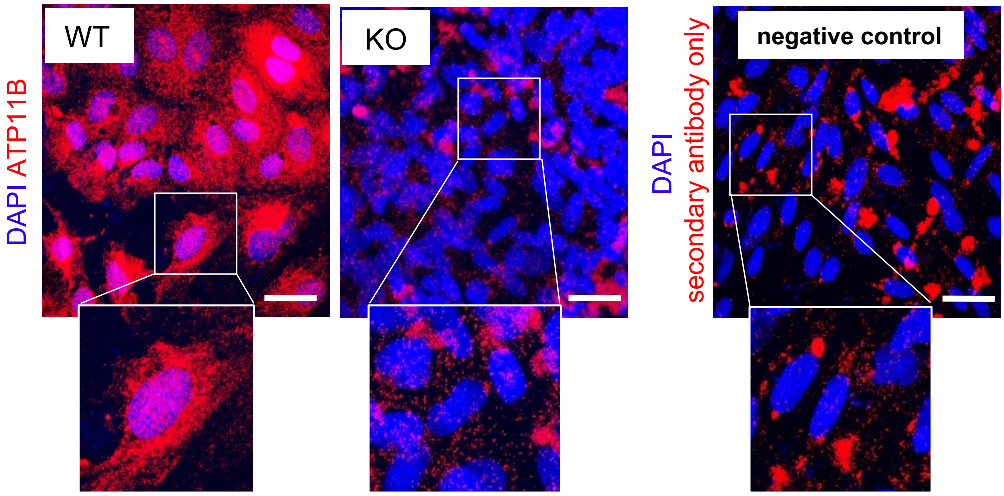

b

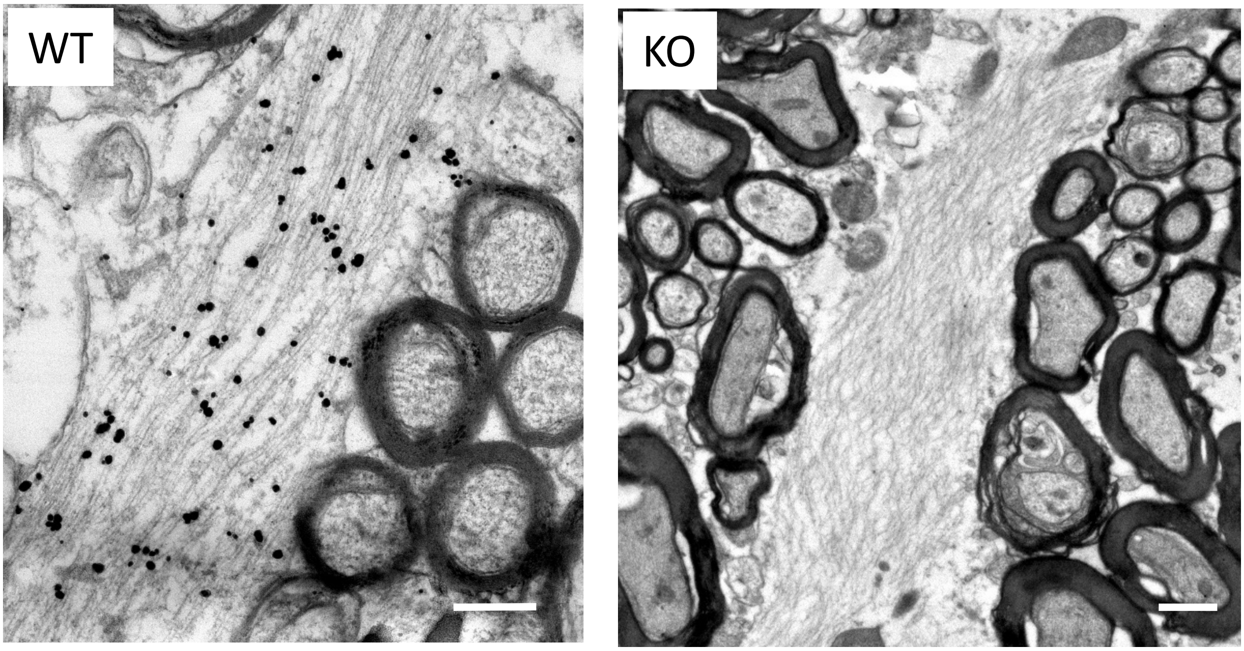

Supplementary Fig. 2 ATP11B antibody specificity

(a) Specificity of ATP11B antibody by immunostaining on cultured brain ECs from WT and KO perinatal animals and secondary antibody only showing background staining. (ATP11B – red, DAPI – blue) Scale bars = 10μm. (b) Specificity of ATP11B antibody by immunoelectron microscopy on WT and KO deep white matter. Filamentous astrocytic processes in the wildtype rat contain many immunogold particles whereas those of the KO animal show no more than minimal background levels. Both micrographs are representative of the labelling seen at the same depth of tissue in 30-week-old animals. Scale bars = 2 μm.

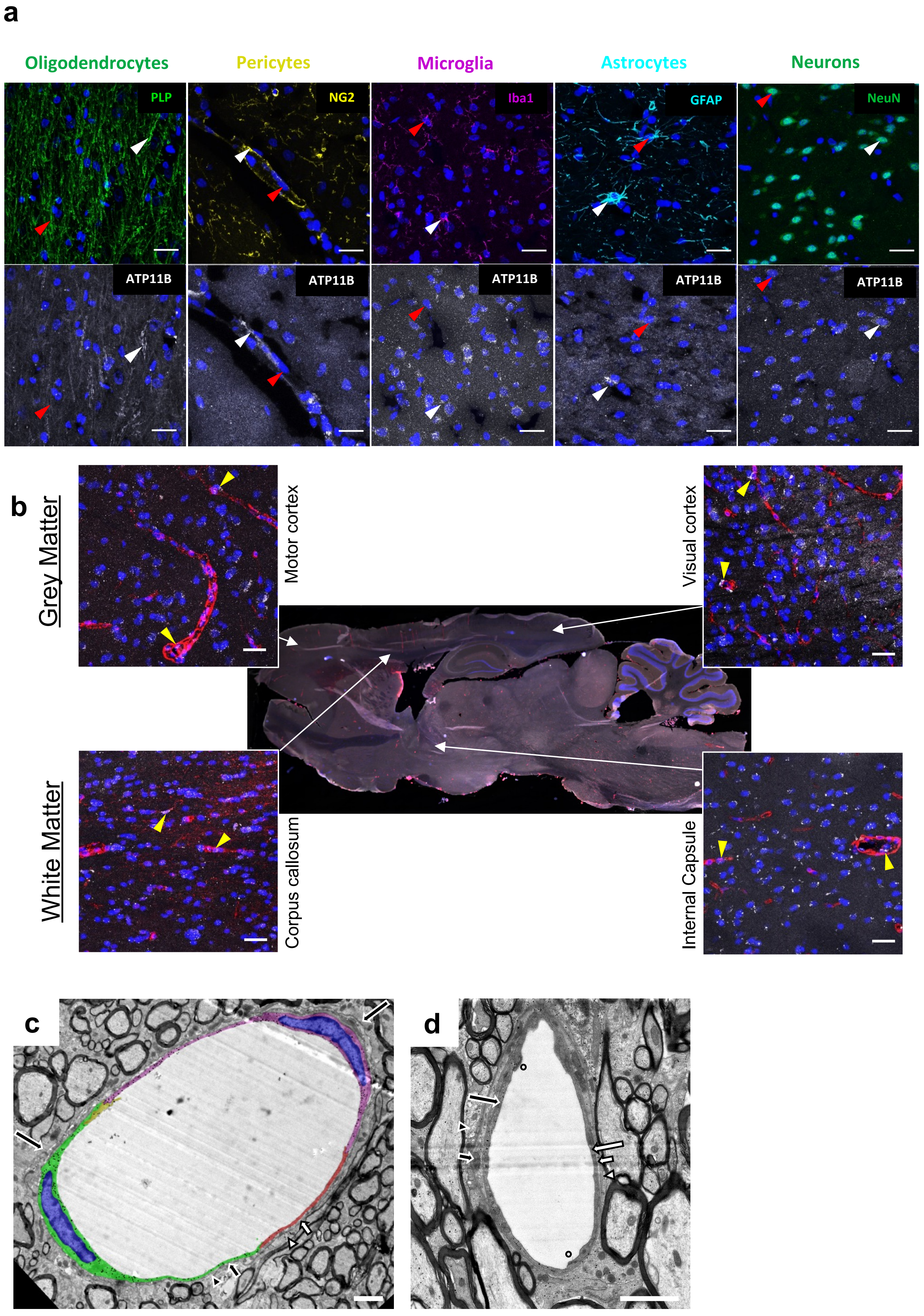
a
Oligodendrocytes
Pericytes
Microglia
Astrocytes
Neurons
PLP
NG2
Iba1
GFAP
NeuN
ATP11B
ATP11B
ATP11B
ATP11B
ATP11B
b
Grey Matter
Motor cortex
Visual cortex
White Matter
Corpus callosum
Internal Capsule
c
d

Supplementary Fig. 3 Heterogeneous expression of ATP11B in brain cells and expression in ECs does not relate to pericyte or astrocyte coverage

(a) ATP11B is expressed heterogeneously by (L-R) oligodendrocytes (PLP+), pericytes (NG2+), microglia (Iba1+), astrocytes (GFAP+) and neurons (NeuN+). Red arrows indicate no ATP11B expression, white arrows indicate ATP11B expression. DAPI nuclear stain – blue. Scale bars = 10μm. (b) ATP11B-positive ECs are found through the brain in white and grey matter. Sagittal section showing location of magnified images of ATP11B (white) and IsoB4 (red) staining (DAPI – blue). Yellow arrows indicate ATP11B-expressing cells. Scale bars = 10μm. (c, d) There is no association between ATP11B expression in ECs and pericyte or astrocyte coverage. (c) In one ATP11B-immunogold stained EM section traced to create the 3D reconstruction in Fig. 1d, adjacent ATP11B^{+} EC1 (green) and ATP11B^{-} EC5 (red) are both surrounded by a similar ATP11B^{-} pericyte layer (small arrows) and ATP11B^{+} astrocytic process (arrowheads). Larger astrocytic processes (large arrows) approach the vessel at the point where the EC layer is thickest (around the nuclei of ECs 1 and 4). Scale bar = 2 μm. (d) Another vessel from the same EM sample as c, showing a smaller vessel, consisting of 2 ECs that meet at tight junctions (circles). The EC on the left (large black arrow) displays multiple ATP11B-gold particles, whereas the EC on the right (large white arrow) shows none. Each is surrounded by layers of pericyte (small arrows) and astrocyte (arrowheads) at the middle and top of the micrograph, and at the bottom of the micrograph. Each EC lacks pericyte coverage but retains astrocytic coverage either side of the tight junction. Scale bar = 2 μm.

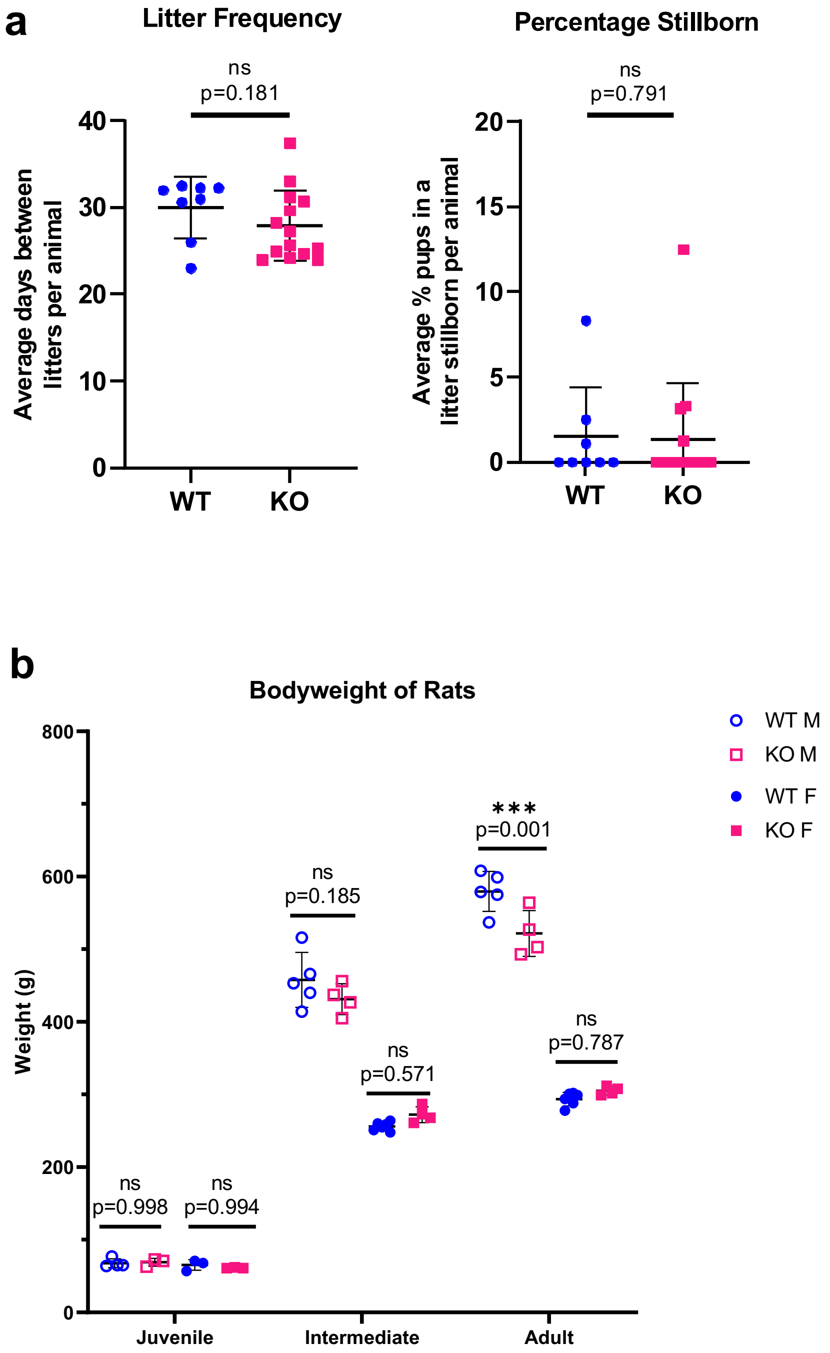

Supplementary Fig. 4. Health of *ATP11BKO* rats

(a) ATP11BKO animals gain weight similarly to WT animals, with sex differences emerging as the animals age. No difference is seen in body weight of developing juvenile animals (Males WT 67.7+/-6.2g KO 69+/-5.3g, Females WT 65.3+/7.4, KO 61.3+/-0.6g) but older males only are significantly lighter (Male WT 579.6+/-27.4g, KO 521.8+/-31.6, Females WT 293.7+/-9.3, KO 305.2+/-5.8g). Animals also breed as normal, with no difference in (b) frequency of litters (Mann Whitney U=36, p=0.181) or (c) percentage of stillborn pups (Mann Whitney U=55.50, p=0.791), calculated as the average per animal having had 3 or more litters. All graphs show mean +/- SD.

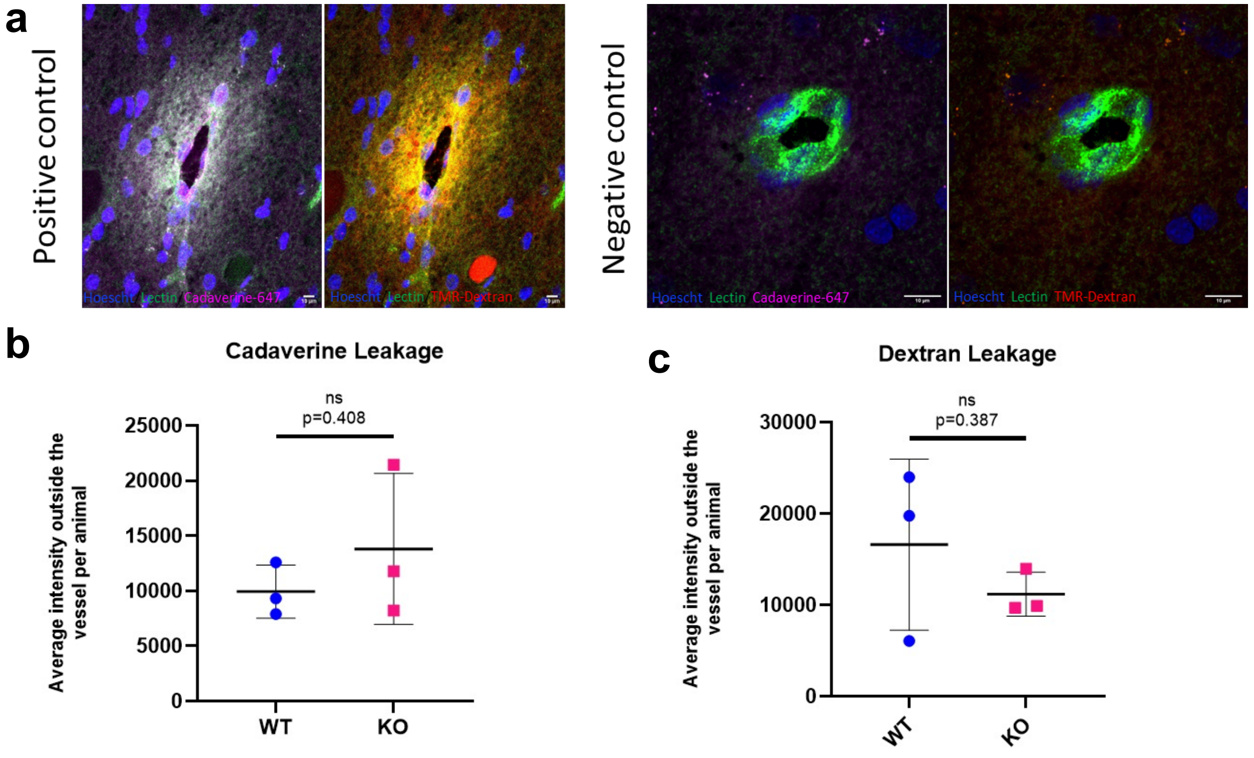

Supplementary Fig. 5. BBB remains intact in juvenile *Atp11b*KO animals

(a) Representative images from the positive (mannitol + tracer) and negative controls (FITC-Lectin only) showing clear leakage around the vessel in the positive control and not in the negative control animal. (b) No difference in cadaverine intensity around vessels in the white matter between WT and KO (t-test, p=0.408, t=0.924, df=4). (c) No difference in dextran intensity around vessels in the white matter between WT and KO (t-test, p=0.387, t=0.970, df=4). All graphs show mean +/- SD.

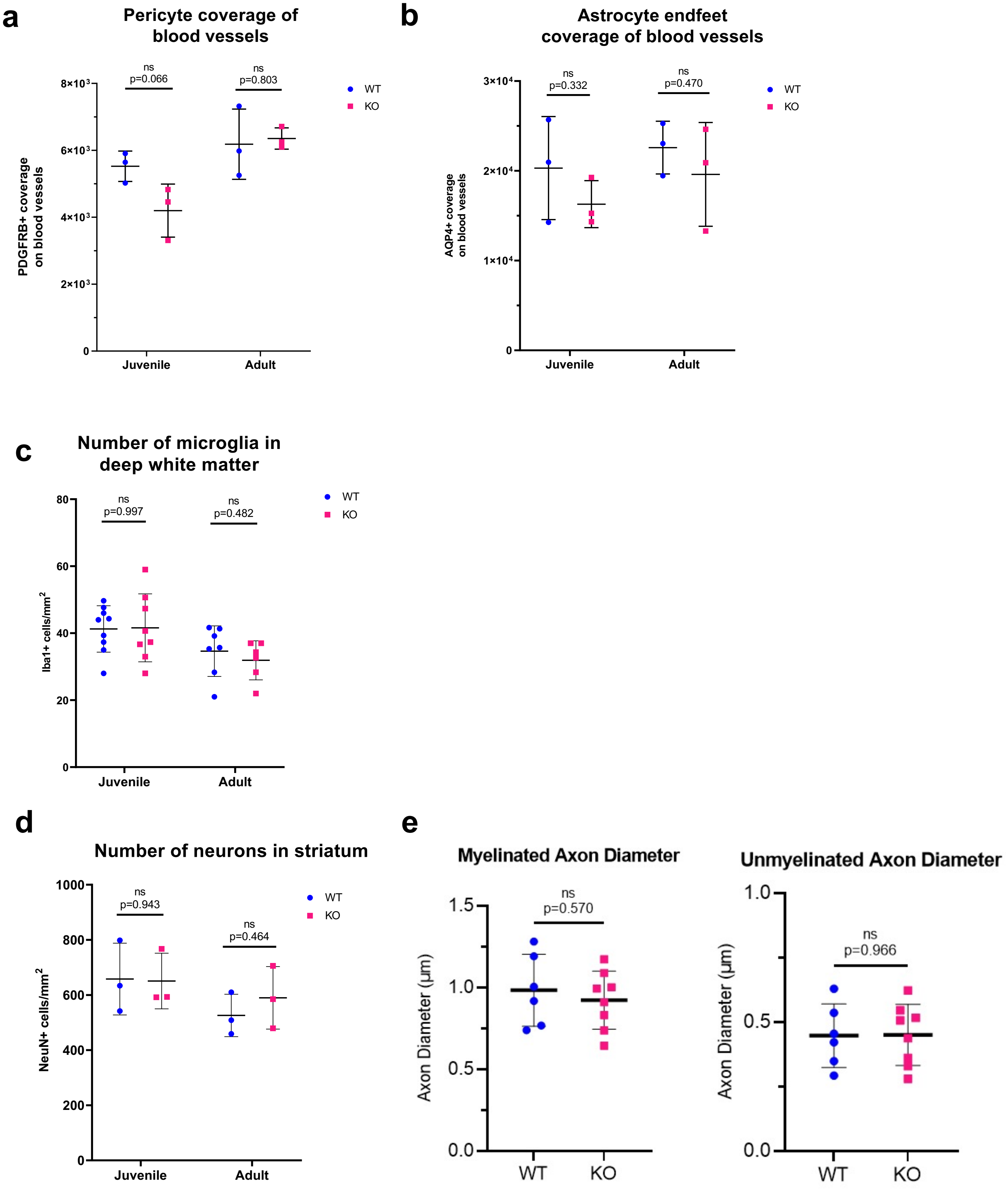
a
Pericyte coverage of blood vessels
PDGFRB+ coverage on blood vessels
8×10³
6×10³
4×10³
2×10³
0
ns p=0.066
ns p=0.803
Juvenile
Adult
WT
KO
b
Astrocyte endfeet coverage of blood vessels
AQP4+ coverage on blood vessels
3×10⁴
2×10⁴
1×10⁴
0
ns p=0.332
ns p=0.470
Juvenile
Adult
WT
KO
c
Number of microglia in deep white matter
Iba1+ cells/mm²
80
60
40
20
0
ns p=0.997
ns p=0.482
Juvenile
Adult
WT
KO
d
Number of neurons in striatum
NeuN+ cells/mm²
1000
800
600
400
200
0
ns p=0.943
ns p=0.464
Juvenile
Adult
WT
KO
e
Myelinated Axon Diameter
Axon Diameter (μm)
1.5
1.0
0.5
0.0
ns p=0.570
WT
KO
Unmyelinated Axon Diameter
Axon Diameter (μm)
1.0
0.5
0.0
ns p=0.966
WT
KO

Supplementary Fig. 6. No changes to other brain cell types seen in *Atp11b*KO animals

Pericyte and astrocyte end foot coverage of blood vessels is not affected by the loss of ATP11B, as measured from images created with multiplex immunostaining. Pixel count of cell-specific marker/area of blood vessel showed (a) no significant difference in pericyte cover as indicated by PDGFRB pixel count/IsoB4+ area (t-tests, juvenile: p=0.066, t=2.517, df=4 ; adults: p=0.803, t=0.266, df=4) and (b) no significant difference in astrocyte endfeet coverage on blood vessels as indicated by AQP4 pixel count/IsoB4 pixel count (t-tests, juvenile: p=0.332, t=1.103, df=4 ; adults: p=0.470, t=0.796, df=4). (c) No significant difference in the number of microglia in the deep white matter (t-tests, juvenile: p=0.997, t=0.078, df=15; adults: p=0.482, t=0.726, df=11). (d) No significant difference in neuronal number in the striatum (t-tests, juvenile: p=0.943, t=0.075, df=4; adults: p=0.464, t=0.808, df=4) or (e) in axonal diameter of myelinated and unmyelinated axons in the white matter between groups at the adult time-point (t-tests, myelinated: p=0.570, t=0.575, df=12 ; unmyelinated: p=0.966, t=0.0442, df=12). All graphs show mean +/- SD.

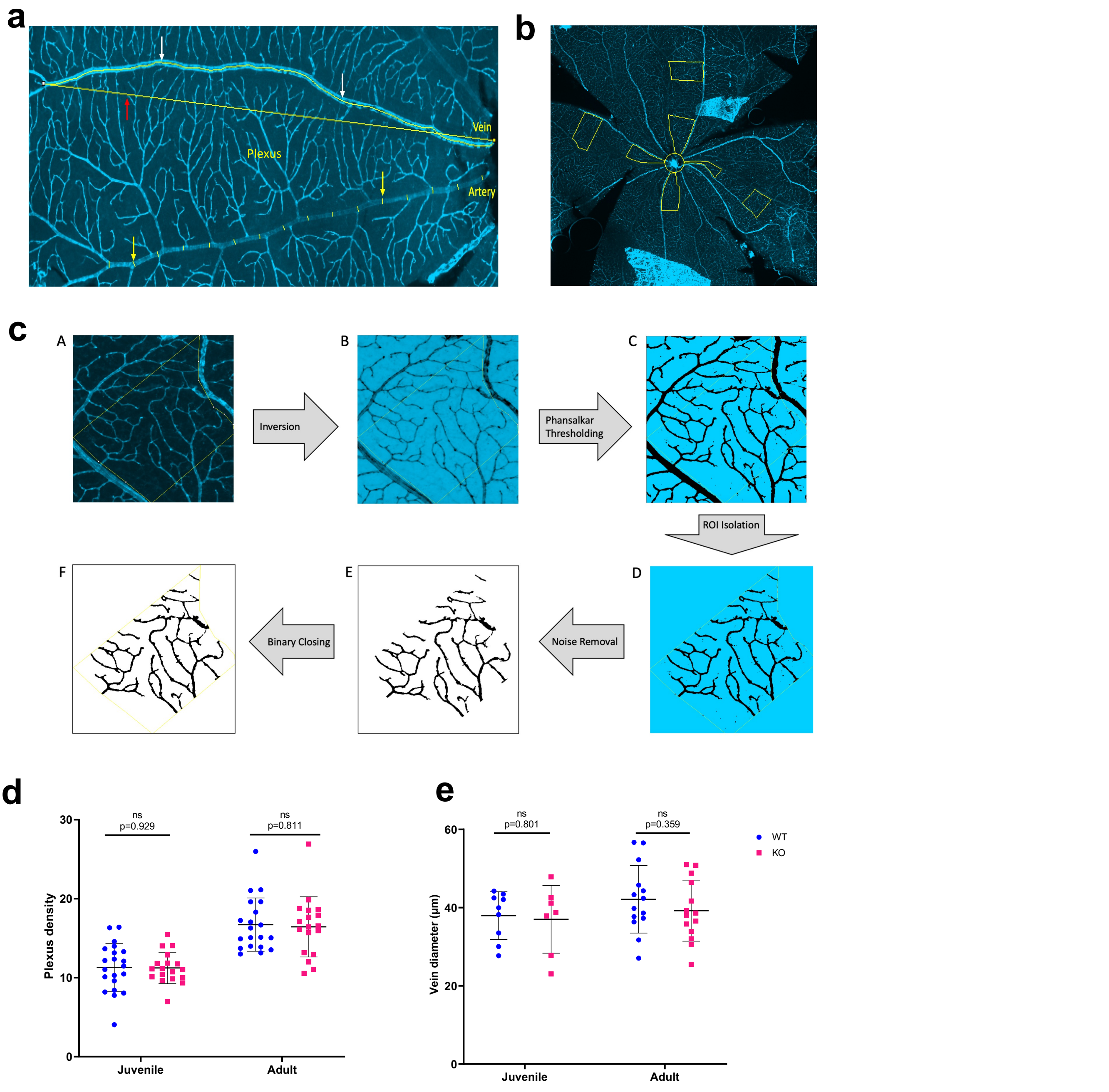

Supplementary Fig. 7 Retinal image ROI selection and additional data

(a) Representative image of tortuosity and diameter measurement in the major vessels. White arrows indicate measurement of total vessel length, red arrow indicates measurement of the Euclidean distance between vessel endpoints, yellow arrows indicate diameter measurements. (b) Representative image of ROI selection. Four proximal ROIs are located centrally, with three distal ROIs outside. A central ring is shown for measurement of the optic disc diameter. (c) Workflow with representative images of a distal ROI at various stages of processing to prepare for analysis. (d) Retinal plexus density is not significantly different between WT and KO animals at either juvenile age (t-test, $p=0.929$, $t=0.089$, $df=37$) or adult age (t-test, $p=0.811$, $t=0.24$, $df=36$). (e) Vein diameter is not significantly different between WT and KO animals at either juvenile age (t-test, $p=0.801$, $t=0.357$, $df=14$) or adult age (t-test, $p=0.359$, $t=0.93$, $df=26$). All graphs show mean +/- SD.

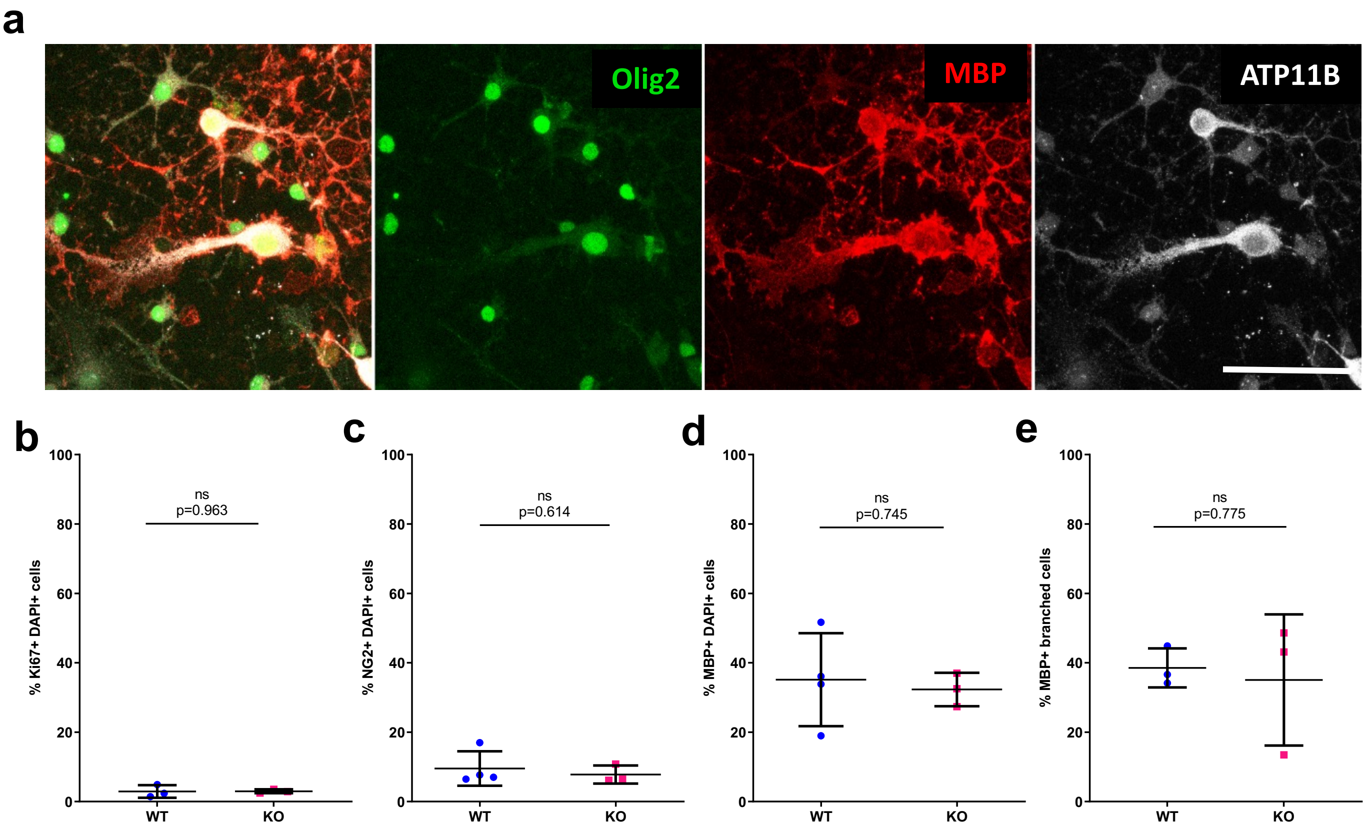

Supplementary Fig. 8 ATP11B expression and proliferation of oligodendroglia

(a) ATP11B is expressed by WT OPCs in culture by immunofluorescence, scale bar 20μm, nuclear OLIG2 in green, MBP in red and ATP11B in grey. Loss of *Atp11b* does not have an effect on OPCs *in vitro* either in (b) number of Ki67+ proliferating cells (t-test, p=0.963, t=0.04888, df=4), (c) number of immature NG2+ cells (t-test, p=0.614 t=0.5380, df=5), (d) number of mature MBP+ cells (t-test, p=0.745, t=0.3442, df=5) or (e) number of branched MBP+ cells (t-test, p=0.775, t=0.3051, df=4). All graphs show mean +/- SD.

a

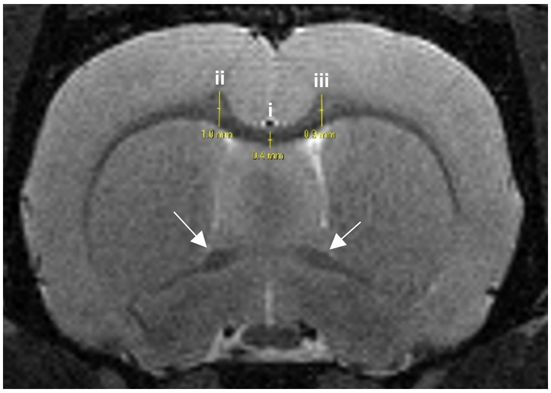

b

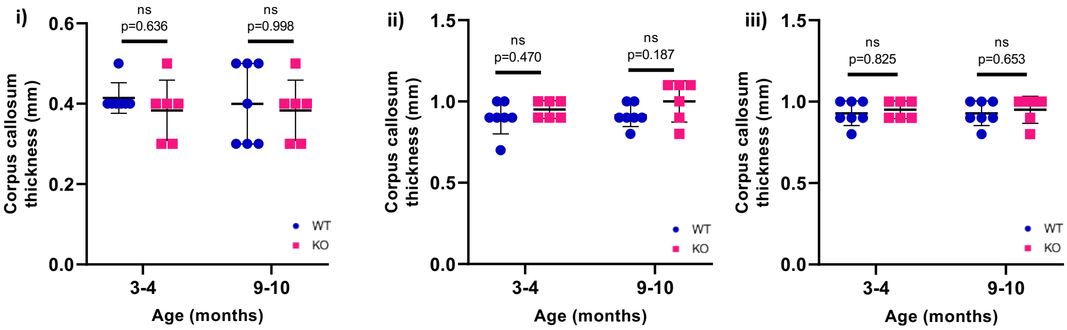

Supplementary Fig. 9 No change in *Atp11b*KO white matter size measurements

(a) Diagram demonstrating corpus callosum measurements on T2-weighted images taken in transverse section just prior to the anterior part of the anterior commissure merging (arrows). Three measurements were taken in positions i, ii and iii of the corpus callosum. (c) No significant difference in corpus callosum thickness between WT and KO at position i) (3-4 months: Mann-Whitney U =15.50, p=0.636 9-10 months: Mann-Whitney U =19, p=0.998; ii) (3-4 months: Mann-Whitney U =15, p=0.470; 9-10 months: Mann-Whitney U =11.50, p=0.187); or iii) (3-4 months: Mann-Whitney U =18, p=0.825; 9-10 months: Mann-Whitney U =17, p=0.653). All graphs show mean +/- SD.

Supplementary Video 1. Heterogeneous expression of ATP11B in ECs of the same vessel.

A 360° rotation of the 3D reconstruction shown in Fig. 1d, showing 5 EC profiles traced and reconstructed in 46 serial sections. Four of the ECs (ECs 1-4, green, yellow, cyan and magenta) show high densities of ATP11B-gold labelling (133-196 particles/ μm^3), whereas EC5 (red) shows a much lower density of gold labelling (14 particles/ μm^3), compared to the level of background gold labelling seen in the nuclei (blue) of ECs 1 and 4 (green/magenta); 2.5 particles/ μm^3. For scale, see Fig. 1d.
